# A semi-automated imaging and analysis pipeline for NET quantification and temporal-profiling of NETosis

**DOI:** 10.3389/fimmu.2026.1753477

**Published:** 2026-03-11

**Authors:** Chloé Landry, Liyuan Wang, Emma Gerber, Chet Elliot Holterman, Dylan Burger

**Affiliations:** 1 Kidney Research Centre, Inflammation and Chronic Disease Program, The Ottawa Hospital Research Institute, Ottawa, ON, Canada; 2 Department of Cellular and Molecular Medicine, Faculty of Medicine, University of Ottawa, Ottawa, ON, Canada; 3 Cell Biology and Image Acquisition Core Facility, Faculty of Medicine, University of Ottawa, Ottawa, ON, Canada

**Keywords:** high throughput analysis, live-cell imaging, machine learning, NETs, neutrophil extracellular traps, quantification pipeline

## Abstract

NETosis is a distinct form of neutrophil cell death involved in innate immunity and characterized by the release of DNA, that when dysregulated contributes to tissue damage and target-organ injury. As our understanding of the mechanisms governing this process continues to advance, there is a growing need for refined tools that can precisely characterize NETosis and enable efficient screening of its modulators. Here, we present a novel live-cell imaging and analysis pipeline for quantifying NETosis in cultured cells. We have generated a CellProfiler pipeline that enables in-depth analysis of cell and NET features, allowing for subsequent characterization and classification of NETosis stages using machine learning. Coupled with high throughput live cell imaging, this approach allows for large-scale and automated tracking of NETosis progression. The pipeline was validated in promyelocytic HL-60 cells differentiated into granulocyte-like cells, as well as primary neutrophils from the bone marrow of mice. We further confirmed dose and stimulus-dependent responses to common stimuli and pharmacological inhibitors of NETosis. As a flexible and scalable high-throughput imaging pipeline, our novel approach allows for the assessment of NETting dynamics and the screening of potential NETosis-modulating agents, which will be instrumental in developing therapies for NET-induced tissue injury.

## Introduction

Neutrophil extracellular traps (NETs) are intricate networks of DNA, citrullinated histones, and granular proteins released during a distinct type of neutrophil cell death known as NETosis (1). While NET formation has evolved as an extension of innate immunity to physically trap and neutralize pathogens, NETs have also emerged as mediators of tissue injury and disease pathogenesis (1). Given the role of NET release and impaired clearing in immune function and immune-mediated tissue injury, more precise methods to study NETosis are needed to elucidate its underlying mechanisms, identify key drivers of its dysregulation, and facilitate drug development and testing (1).

NETs can accumulate in various organs and within the vasculature, where they trap and kill bacteria and contribute to thrombus formation (2). The abrasive nature of NET components, which include DNA, granular protein and histones, renders them cytotoxic to surrounding cells (3).

The detection of cell-free DNA is a widely used measure of NET content, despite its inability to discriminate NETs from necrotic DNA release (4). While this is the simplest way of assessing the presence of NETs, it is not sensitive or precise enough to apply as a method to investigate NETosis mechanisms. Other established methods include immunofluorescent or flow cytometry quantification of labelled NET components, typically combining DNA dyes with NET-associated markers such as neutrophil elastase and citrullinated histone H3 to confirm NET identify (5). Although they provide increased specificity, such approaches tend to be expensive and tedious and require sample manipulation steps that risk disrupting the delicate NET structures (6). As such, a standardized method for detection and quantification of NETs has yet to be established in the research community.

Immunofluorescence imaging is widely employed in the study of NETosis, as it enables visualization of NET structures and components. Advances aimed at streamlining methodology for NET quantification in fluorescence microscopy images have provided tools to facilitate image analysis, yet the heterogeneity of NETs poses a challenge for establishing comprehensive and reproducible analyses (5, 7). Current pipelines for analyzing NET release *in vitro* largely rely on measuring the surface area of DNA stained with cell-impermeant fluorescent DNA dyes (i.e. SYTOX) or a chromatin antibody, reporting on NET counts or area, and inferring the percentage of NETting cells by normalizing to total cell count (8). However, NETs are defined by varying DNA staining features across different methods. Some studies quantify NETs as the total DNA-positive area, while others use metrics such as the size or integrated density of DNA-positive regions to define NETs (8, 9), leading to inconsistent data interpretation. Although some approaches are more comprehensive and incorporate multiple DNA stain features (10), they still rely exclusively on a limited set of predefined DNA staining metrics, a constraint that can reduce specificity and risk overfitting.

Application of machine learning algorithms for single-cell NET labeling can enable automated thresholding to measured parameters or the learning of classification boundaries directly from image data using convolutional neural networks (11, 12). While these methods increase specificity and reduce bias from manual thresholding, to date they have been employed with limited staining panels or narrowly focused deep learning algorithms that hinder the development of generalizable classifiers capable of accurately resolving distinct phenotypes and distinguishing NETosis from other forms of cell death.

Many factors contribute to phenotypic variability among NETting cells, making it challenging for stringent models or fixed parameters to capture the full spectrum of NETosis across cells of different origins. More granular characterization of NETosis has shown that it represents a distinct form of programmed cell death that proceeds through a temporally ordered sequence of molecular and morphological changes (13, 14). Accordingly, discrete stages of neutrophil activation and NET release have been described across multiple experimental platforms, reflecting a progression from early cellular activation to chromatin decondensation, and extracellular DNA release (14–16). While the nomenclature and granularity of intermediate stages vary across methods, most frameworks capture this sequential progression, which was first described as a temporally ordered three-phase process (P1–P3) by Fuchs et al. (15).

While this inherent heterogeneity complicates analysis, it also provides valuable insight into the mechanisms governing NET formation. Imaging-based approaches have been developed to resolve these features, often relying on high-resolution microscopy and complex staining panels (13, 14). In contrast, applying generalized threshold-based methods to such heterogeneous phenotypes can be fundamentally limiting, as it often requires subjective parameter selection and relies on a small set of predefined features that may be insufficient to capture the full complexity of NETosis (16).

To address this, we present a comprehensive experimental design from live-cell imaging to machine learning-assisted pixel and object classification for broad-spectrum identification of NETs and NETosis stages, with distinction from other types of cell death. This pipeline integrates phase-contrast imaging with DNA and Annexin V staining, a phosphatidylserine-binding probe that detects early stages of cell death, to enable detailed analysis of individual cells and associated NET areas while allowing discrimination of NETotic cells from apoptotic and necrotic cells. Machine learning is then applied to train a Random Forest classifier that leverages distinguishing features to classify NETosis phenotypes defined according to established morphological criteria, corresponding to stages of cell spreading, nuclear disintegration, and NET release. The classifier is adapted to each image dataset, providing a quality control step that ensures appropriate discriminative features and thresholds for accurate classification of NETosis stages across conditions.

Briefly, the spread stage is characterized by a flattened cell morphology with a compact nucleus, the disintegrated nucleus stage by dispersion of DNA throughout the cytoplasm, and NETosis by extrusion of decondensed DNA into the extracellular space, either as a diffuse cloud or through a membrane pore, frequently accompanied by Annexin V positivity at the cell perimeter. Cells lacking detectable fluorescent signal were classified as negative, while Annexin V– and DNA-positive cells exhibiting a shrunken and irregular morphology were classified as dead.

**Figure 1:**
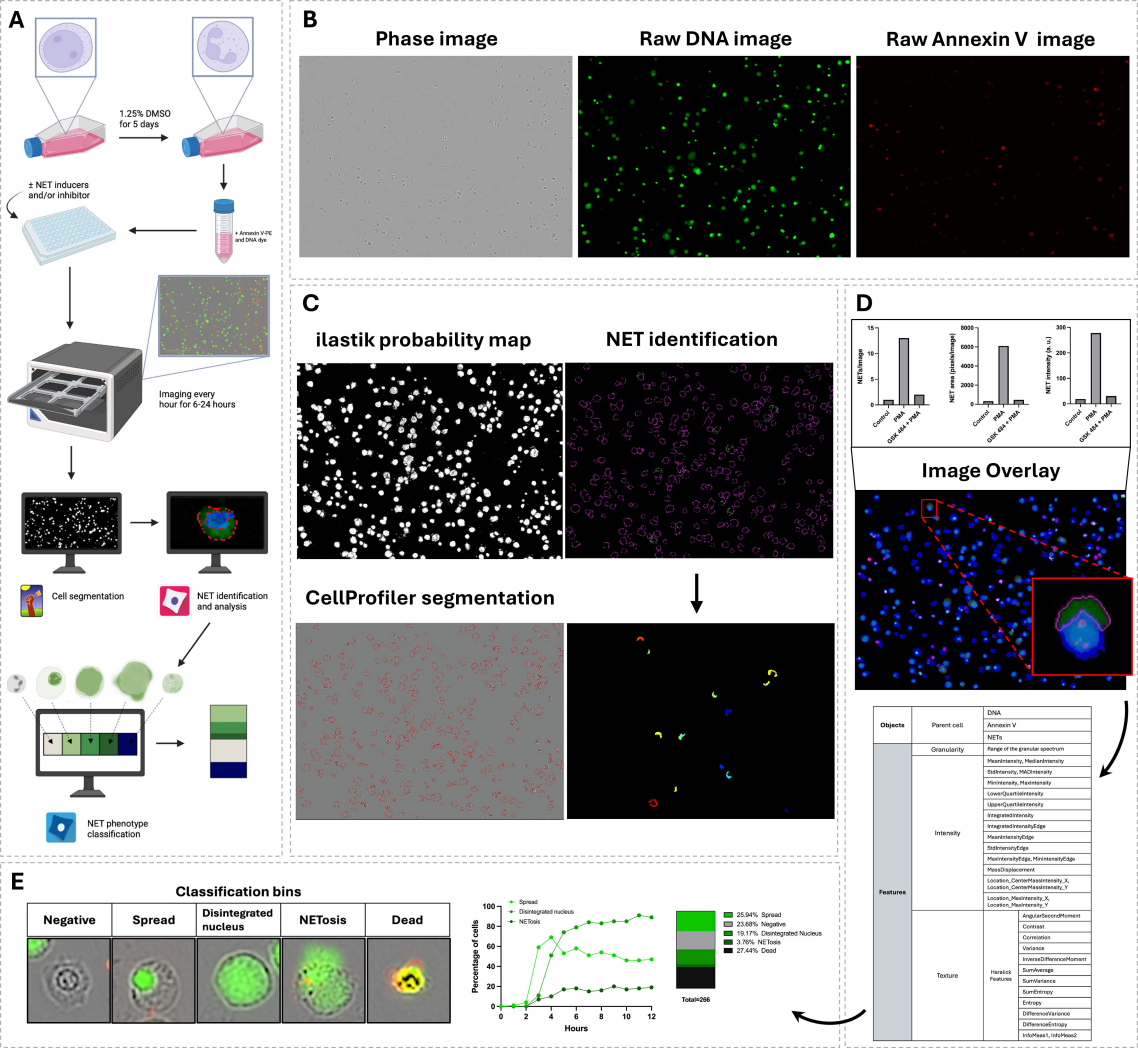
Overview of the experimental workflow and analysis for NETosis quantification. **(A)**, Schematic of the workflow with differentiated HL-60 cells. Created in BioRender (https://BioRender.com/9rvj2i1). **(B)**, Live-cell imaging is performed every hour for 12 hours using three channels: Phase contrast, green fluorescence (DNA), and red fluorescence (Annexin V). **(C)**, A segmentation probability map is generated from each phase image using *Ilastik*. All images, including the segmentation probability map, are processed in a custom *CellProfiler* pipeline to identify individual cells, DNA and Annexin V. NETs are defined as regions of green fluorescence located outside of the cell body, and all identified objects are mapped back to the parent cell. **(D)**, NET count, area, and fluorescence intensity are measured per image. For each individual cell and its associated DNA, Annexin V, and NET structures, morphological (e.g., shape, size), textural, granular, and intensity-based features are extracted and saved as a CSV file. **(E)**, Extracted object-level features are used by *CellProfiler Analyst* to train a Random Forest classifier on user-annotated objects. The classifier is then used to categorize cells into different NETosis stages, enabling quantitative assessment of NETosis dynamics and phenotype distributions over time.

Together, this pipeline provides a framework for high-throughput live-cell imaging and semi-automated quantification of NETosis dynamics over time in isolated neutrophils (**Figure 1A**). It enables real-time tracking of NET release and progression through NETosis stages, providing detailed insight into both temporal patterns and phenotypic features. By combining phase-contrast imaging with fluorescence detection of extracellular DNA and Annexin V, the assay captures key morphological and functional changes of NETosis (**Figure 1B**). This information helps distinguish NETting cells from dying cells, while detecting a broader range of NET phenotypes than traditional assays. The pipeline integrates open-source tools: *ilastik* for pixel classification (17), *CellProfiler* for object segmentation (18), and *CellProfiler Analyst* (CPA) for downstream classification of NETosis stages (19) (**Figure 1C**). Total-image NET characteristics such as count, area and fluorescence intensity can be quantified to provide a broad assessment of NETosis (**Figure 1D**). Additionally, single-cell features obtained from CellProfiler are used to train classifiers in CPA, enabling the assignment of each cell to a NETosis stage and quantification of stage distributions over time (**Figure 1E**). This method enables mechanistic studies by revealing when and how specific treatments alter NETosis, and its 96-well format supports large-scale screening of NETosis modulators (17).

This pipeline was initially established using promyelocytic HL-60 cells differentiated to neutrophil-like cells to precisely define NET areas and develop an algorithm to distinguish the various NETosis stages. It was subsequently adapted and validated in mouse neutrophils isolated from bone marrow. Leveraging the established role of peptidylarginine deiminase 4 (PAD4) as a key mediator of NET release (20), we validated both the specificity of detected NET areas and its resulting ability to identify NETosis inhibition. Accordingly, NETosis was assessed in differentiated HL-60 cells treated with a selective PAD4 inhibitor, and in neutrophils from *Padi4*^-/-^ mice, which are incapable of forming NETs (21).

## Materials and methods

### NETosis model for pipeline development

The promyelocytic HL-60 cell line (ATCC), differentiated into granulocyte-like cells, was used for the development and validation of the CellProfiler analysis pipeline and subsequent single-cell NETosis stage classification with CellProfiler Analyst (CPA). Cells were cultured in Iscove’s Modified Dulbecco’s Medium (IMDM) with phenol red (Thermo Fisher Scientific), supplemented with 10% fetal bovine serum (FBS, Wisent) and 1% penicillin-streptomycin. Differentiation was induced by transferring cells into complete culture medium containing 1.25% dimethyl sulfoxide (DMSO) at a final density of 4x10^5^ cells/mL, followed by incubation at 37 °C with 5% CO_2_ for 5 days (22). After incubation, cell viability was assessed using Trypan Blue, and differentiated cultures with ≥75% viability were used for the assay. The NET assay was repeated five times with independent batches of differentiated cells, with three replicated wells per treatment: untreated, treated with 200 nM phorbol 12-myristate 13-acetate (PMA) (Millipore-Sigma), or pre-treated with 20 µM GSK484 (Cayman Chemical), a selective PAD4 inhibitor (20), for 30 minutes prior to PMA stimulation.

### Pharmacological modulation of NETosis dynamics

The above-described neutrophil-directed HL-60 cells were used to compare NETosis dynamics between different inducers and assess the contribution of PAD4 with both PMA and ionomycin stimulation. The NET assay was repeated five times with independent batches of differentiated cells, with three replicated wells per treatment. Cells were either left untreated or treated with 0.2, 2, 20, or 200 nM PMA or 5 µM ionomycin (Millipore-Sigma). In separate wells, cells were pre-treated with 20 µM GSK484 for 30 minutes prior to either 200 nM PMA or ionomycin stimulation.

### Validation with primary bone marrow neutrophils

All animal work was approved by our institutional ethics committee. Wild-type (WT) and peptidylarginine diminase 4-deficient (*Padi4*^-/-^) (23, 24) male mice on an FVB background (12- to 14-weeks of age) were used for bone marrow neutrophil isolation (3 WT mice and 4 *Padi4*^-/-^ mice), according to a previously described protocol (25). Briefly, muscle and connective tissue were removed from the femoral bone, and the condyles, the patella, and the epiphysis were excised with scissors to expose the metaphysis. The femur was placed knee end down in a 0.5 mL microcentrifuge tube nested in a 1.5 mL microcentrifuge tube and centrifuged at ≥10,000 x g for 15 seconds to collect the bone marrow. Bone marrow cells were processed for negative immunomagnetic selection of neutrophils using the Miltenyi Biotec mouse Neutrophil Isolation Kit, in which all non-neutrophil populations were depleted by incubation with a cocktail of biotinylated antibodies and anti-biotin magnetic microbeads, followed by separation with a magnetic column. Typical yields were ∼ 2–9 x 10^6^ neutrophils per femur. Isolated neutrophils were subjected to the live-cell imaging NET assay and either left untreated or stimulated with 100 nM PMA, in three well replicates per condition.

### Assay preparation

To prepare for NETosis imaging, each well of a 96-well flat bottom culture microplate (Corning) was coated with 0.1 μg/mL fibronectin from bovine plasma (Millipore Sigma) in PBS and incubated at room temperature for 1 hour. The assay culture medium consisted of serum-free IMDM (IMDM, phenol red-free; Thermo Fisher Scientific) supplemented with 250 nM Incucyte^®^ Cytotox Green Dye (Sartorius) and 30 ng/mL PE Annexin V (Biolegend). Neutrophils were resuspended in this medium and seeded at a density of 20,000 cells/well. Treatments were added to a final volume of 200 mL per well.

### Live-cell imaging

Images were acquired using the Incucyte^®^ S3 Live-Cell Imaging and Analysis System at 20x magnification in the green, red, and phase contrast channels. For each well, 2–3 fields of view were captured at a frequency of one image per hour over a total duration of 12 or 24 hours. Default acquisition settings were used, with exposure times of 300 ms for the green channel and 400 ms for the red channel, and standard focus offsets applied by the Incucyte software (v2023A). The imaging system was maintained at 37 °C with 5% CO_2_ throughout the experiment. Following image acquisition, uncalibrated images were exported as 8-bit PNG files for the phase-contrast channel and as 16-bit TIFF files for the green and red fluorescence channels.

### ilastik cell segmentation

To enable robust cell segmentation in CellProfiler, phase-contrast images were processed in ilastik using a pixel-level Random Forest classifier trained to distinguish cells from background, independently of the downstream object-level classifiers used for NETosis stage classification. Phase-contrast images were first processed in ilastik (v.1.4.0;https://www.ilastik.org/) using the Pixel Classification workflow to generate segmentation probability maps (17). Separate workflows and segmentation algorithms were generated for each of the cell types (i.e. neutrophil-directed HL-60 cells and mouse bone marrow neutrophils). For each workflow, 5–9 representative images spanning all treatment conditions were selected as training data. All multi-scale image features were enabled for pixel characterization, including smoothed pixel intensity, edge filter, and texture descriptors. Three categories of image filters are provided in ilastik, including color/intensity, edge, and texture features. For filters applied at multiple scales, each scale corresponds to the Gaussian smoothing (sigma) applied prior to feature extraction, with larger sigma values (e.g., σ = 10) capturing broader neighborhood information compared to smaller values (e.g., σ = 0.3). Training images were manually annotated with sparse labels for the two intended classes, *Cells* and *Background*. Using ilastik’s Live Update function, pixels were iteratively annotated until accurate segmentation was achieved, capturing all cell morphologies while excluding background. Each pixel was assigned a feature vector comprising values for each selected image feature, used to train a Random Forest classifier (Vigra’s RandomForestClassifier from the C++ Python for image-processing and machine-learning library). The trained classifier assigned each pixel to a class based on these features and annotations, producing semantic segmentation probability maps for each label. Batch processing was then used to apply the classifier to all unseen images. Resulting probability maps were exported as 32-bit multi-channel TIFF images and imported into CellProfiler, where pixel-probability thresholding was used to generate binary masks for segmentation.

### CellProfiler image processing pipeline

Images were processed using CellProfiler™ cell image analysis software (v4.2.6; https://cellprofiler.org) for segmentation of cells, DNA, Annexin V and NETs, as well as for the extraction of morphological and quantitative object features from phase-contrast, green fluorescence, and red fluorescence images (18). Cell segmentation was facilitated by ilastik-generated probability maps, which were imported into CellProfiler and applied to the corresponding phase-contrast images. This pipeline enabled broad NET quantification by extracting image-level metrics, including NET counts, total area, and fluorescence intensity from the segmented extracellular DNA regions. All resulting object feature measurements were exported as a database file compatible with CPA for subsequent single-cell classification.

#### Metadata

Image metadata were extracted from folder and file names to provide CellProfiler with information on the imaging channel, well, field of view, and time point for each image. Accordingly, folders were organized named by image type (i.e. Phase, Green, Red, and Probability) and images were saved using the default Incucyte naming format: *Plate_Well_Image_Day_Hours_Minute.* Metadata fields were extracted in CellProfiler using a regular-expression–based parsing approach, with full details provided in **Supplementary Methods S1**. To incorporate per-well treatment information, a separate CSV file was created listing each well alongside its associated treatment and uploaded using the *Import from file* metadata extraction method in CellProfiler.

#### Probability maps

The ilastik probability maps were imported into the pipeline as multi-channel RGB images, in which each channel represents the per-pixel probability of the Cells and Background classes. The CellProfiler *ColorToGray* module was used to extract the channel corresponding to the cell class, generating a single grayscale probability image for downstream segmentation. In these maps, background pixels have low intensity values, and pixels classified as cells appear progressively brighter with increasing classification certainty, enabling CellProfiler to interpret pixel intensities as probability values for segmentation.

The grayscale probability maps were smoothed using the *EnhanceOrSuppressFeatures* module (Suppress mode, feature size=10 pixels) to reduce small-scale noise and intensity variations.

The *IdentifyPrimaryObjects* module was used to segment cells based on the intensity values of ilastik-identified ROIs. Cells were identified as white regions ≥15 pixels that did not touch the image border. Adaptive Otsu thresholding (two classes) was applied with a threshold correction factor of 0.3 and a 50-pixel adaptive window. A 10-pixel smoothing filter was used to distinguish clumped objects by shape, with dividing lines determined by intensity gradients. Internal holes within segmented objects were filled.

Segmented cell borders were overlaid on the corresponding phase-contrast images using the *OverlayOutlines* module as a quality control step to validate segmentation accuracy during pipeline optimization.

#### Green fluorescence images

For DNA segmentation, the green fluorescence images were first log-transformed (base 2) using the *ImageMath* module to enhance low-intensity regions and facilitate the detection of faint DNA signals.

All true DNA areas were segmented using a separate *IdentifyPrimaryObjects* module applied to the processed green fluorescence images. Objects ≤1,000 pixels that did not touch the image border were included. Adaptive Otsu thresholding (two classes) was applied with a threshold correction factor of 0.8 and a 50-pixel adaptive window. Intensity threshold limits were optimized per dataset to account for background variation, with the lower inclusion limit ranging from 0.005-0.03. A 10-pixel smoothing filter was used to distinguish clumped objects by shape, with dividing lines determined by intensity gradients. Internal holes within segmented objects were filled.

DNA signals were retained for downstream analysis only when overlapping a segmented cell to ensure consistency with cell segmentation, which excluded cells touching the image border. Object relationships were defined using the *RelateObjects* module, with cells designated as parent objects and DNA regions as child objects. The filtered object set was linked to parent cells using a second *RelateObjects* step, after which all separate DNA objects were merged per-parent cell using the *SplitOrMergeObjects* module. These merged objects were then related back to their parent cells in a final *RelateObjects* step.

#### Red fluorescence images

Prior to Annexin V segmentation, the red fluorescence images were processed using the *EnhanceOrSuppressFeatures*module to enhance speckles with a feature size of 20 pixels, a characteristic size of Annexin V signals, thereby improving their distinction from background noise.

Annexin V objects ≤1,000 pixels that did not touch the image border were segmented using a third *IdentifyPrimaryObjects* module. Adaptive Otsu thresholding (three classes, with middle intensity classified as foreground) was applied on a smoothed image (default smoothing: 1.3488) with threshold bounds set between 0.0002-1, a correction factor of 1, and a 50-pixel adaptive window. Clumped objects were distinguished based on shape using the smoothed image automatically generated by CellProfiler, with dividing lines determined by intensity gradients. Internal holes within segmented objects were filled.

Annexin V signals were retained for downstream analysis only when overlapping a segmented cell to ensure consistency with cell segmentation, which excluded cells touching the image border. Object relationships were defined using the *RelateObjects* module, with cells designated as parent objects and DNA regions as child objects. The filtered object set was linked to parent cells using a second *RelateObjects* step, after which all separate Annexin V objects were merged per-parent cell using the *SplitOrMergeObjects* module. These merged objects were then related back to their parent cells in a final *RelateObjects* step.

#### NET segmentation

To enable NET segmentation, the *MaskObjects* module was applied with the per-cell unmerged DNA objects set as the objects to be masked and the segmented cells as the masking objects. The mask was inverted to retain only DNA regions outside the cell borders.

Segmented NET objects were expanded by 1 pixel using the *ExpandOrShrinkObjects* module to ensure overlap with their parent cells, enabling subsequent relational mapping with the *RelateObjects* module.

The area of each individual NET object was measured using the *MeasureObjectSizeShape* module, after which the *RelateObjects* module was applied to filter objects by size (≥150 pixels) and to keep only those associated with at least one parent cell.

The filtered NET objects were linked to parent cells using a second *RelateObjects* step, after which all disconnected NET objects were merged per-parent cell using the *SplitOrMergeObjects* module. These merged NETs were then related back to their parent cells in a final *RelateObjects* step.

#### Image masks

Prior to object measurement and data extraction steps, all identified objects (i.e. cells, merged DNA, merged Annexin V, and merged NETs) were applied as masks on the phase-contrast, green fluorescence, and red fluorescence images using a series of *MaskImage* modules, to enable data extraction for each object across all imaging channels.

#### Object feature data extraction

A series of object measurements were performed to characterize the morphological and compositional features of each object, with intensity and texture metrics extracted across all imaging channels to assess cross-channel signal associations.

The granularity spectrum of each object was measured using the *MeasureGranularity* module, which applies a series of top-hat filters with structuring elements sized 1–16 pixels to quantify texture coarseness at different spatial scales.

Several intensity features were extracted from each object across all imaging channels using the *MeasureObjectIntensity* module: maximum intensity, minimum intensity, mean intensity, median intensity, lower quartile intensity, upper quartile intensity, intensity standard deviation, and intensity median absolute deviation (MAD).

The spatial distribution of signal intensities within each DNA and NET object was quantified using the *MeasureObjectIntensityDistribution* module, with the edge of the associated cell (periphery) treated as the radial reference point and four radial bins applied. This module reports the fraction of total object intensity at each radius, expressed as mean fractional intensity and the coefficient of variation of intensity within each concentric ring.

Area and shape features are extracted from the objects using the *MeasureObjectSizeShape* module, including area, volume, perimeter, eccentricity, radius, and Zernike shape features.

The degree and nature of texture within objects were assessed using the *MeasureTexture* module, with quantifies object roughness or smoothness by analyzing the distribution of pixel intensities over 256 greyscale levels and calculating Haralick features, including contrast, correlation, variance, and entropy.

To report image-level NET features, the total NET area was measured using the *MeasureImageAreaOccupied* module, while NET intensity features were quantified from the green fluorescence images using the*MeasureImageIntensity*module, including total fluorescence intensity for comparison between treatment groups.

Overlay images highlighting the cells and segmented objects were generated for visualization of analysis output. The phase-contrast, green fluorescence, and red fluorescence grayscale images were converted to RGB format using the *GrayToColor* module, with cells assigned to the blue channel, DNA to the green channel, and Annexin V to the red channel. The *OverlayOutlines* module was then used to outline the outer borders of NETs in fuchsia on the RGB images, which were then saved as 8-bit integer TIFF files using the *SaveImages* module.

All extracted image- and object-level data were exported as an SQLite database file using the *ExportToDatabase* module, with a single object table created for seamless integration with CPA. A CellProfiler properties file was also generated at this stage to enable subsequent single-cell classification with CPA, specifying *object* as the classification type and defining cells as the individual object locations. Finally, all images were batch processed.

### CellProfiler pipeline generalizability and adaptation

The pipeline is applicable across datasets but may require adjustment of segmentation parameters to account for differences in cell size, imaging resolution, and image contrast. These parameters are defined in the *IdentifyPrimaryObjects* module, where expected object size ranges, minimum object size, and intensity thresholds are specified in pixel units. In this study, images were acquired using an Incucyte S3 microscope with a 20×/NA 0.45 objective (0.62 µm per pixel), such that a minimum object size threshold of 15 pixels (≈5.8 µm²) was used to exclude non-cellular debris. When applying the pipeline to other microscopes or cell types, size parameters should be rescaled according to pixel size and expected cell dimensions. Threshold correction factors may also require adjustment depending on phase-contrast image quality. CellProfiler’s visualization tools and summary statistics facilitate rapid parameter optimization of these parameters during pipeline adaptation.

### Image-level data extraction and analysis

A *per_image* table was generated as part of the database file, containing all image-level extracted data. For broad NET assessment at this stage, the metrics *Image_Count_NETs_merged* (NET count per image), *Image_AreaOccupied_NETs_merged* (NET area in pixels per image), and *Image_Intensity_TotalIntensity_DNA_NETs_merged* (NET intensity (A.U.) per image) were used. Outliers were identified and removed on a per-experiment, per-treatment, and per-time-point basis for each metric using the interquartile range (IQR) method, implemented in Python (v3.11) using pandas. Data points outside were excluded from downstream analyses. Overall, 28% of FOVs were removed. Distribution of removed FOVs was verified to be approximately uniform across wells to avoid introducing differential sampling bias. Remaining values were normalized to the total cell count per image (*Image_Count_Cells*), averaged across all fields of view (*Image_Metadata_Image*), and stratified by treatment (*Image_Metadata_Treatment*) and time point (*Image_Metadata_Hours*).

A global Kruskal-Wallis test was performed to assess overall differences in image-level metrics across treatments, followed by pairwise Mann-Whitney U tests with Holm correction for multiple comparisons. Statistical significance was defined as p < 0.05 after correction. This threshold was applied to all subsequent statistical analyses unless otherwise specified.

For bone-marrow neutrophil image sets, NET image-level data were not quantified at this stage due to an unexpected non-viable cell phenotype, in which extruded DNA could be misclassified as NETs.

### Comparison to hand counts

NET counts from 9 representative images (3 per treatment group) were quantified by two independent manual annotators and by the CellProfiler pipeline. Agreement and bias between methods was assessed using Kruskal-Wallis tests with appropriate *post-hoc* corrections, and intraclass correlation coefficients (ICC2 and ICC3) were reported to evaluate inter-rater absolute agreement and consistency.

### Single-cell NETosis stage classification

Each cell identified during CellProfiler segmentation was classified into one of the predefined NETosis stages (negative, dead, spread, disintegrated nucleus, or NETosis) using the machine-learning classifier in CellProfiler Analyst™ (CPA, 3.0.4; https://cellprofileranalyst.org) (19). Class definitions followed previously published nomenclature and morphological criteria (14). An initial Random Forest classifier was trained using manual annotation of 100–200 cells per phenotype category from segmented cell objects randomly sampled by CPA across all experimental images (**Supplementary Figure 4****).** For subsequent datasets, this classifier was used to pre-classify objects and guide retraining, after which a comparable number of cells per category were reviewed and refined prior to updating the classifier.

Features extracted from segmented objects (per_object table) in the CellProfiler database file and its included features, as described in the CellProfiler image analysis pipeline above, were used to train the algorithm and identify informative features for distinguishing cell phenotypes. Overall classifier accuracy and per-class accuracy were evaluated using the CPA confusion matrix, which reports the proportion of manually labeled cells correctly predicted by the algorithm. Classifier performance was assessed using 5-fold cross-validation in CPA, with an 80/20 train-test split ratio applied to the manually annotated object set in each fold. Accuracy metrics, including precision, recall, and F1-score, were calculated and reported as the average across all 5 folds. Each dataset was batch processed separately to account for experiment-specific phenotypic variations and to avoid forcing a single classifier across heterogeneous conditions. Following cell classification, an image-level per-class cell count table (HitTable) was generated in CPA. Outliers were identified and removed on a per-experiment, per-treatment, and per-time-point basis within each phenotypic class using the IQR method, implemented in Python (v3.11) using pandas. Following IQR-based filtering, 28% of FOVs were excluded due to extreme values. Exclusion rates were comparable across wells. Data points outside were excluded from downstream analyses. The remaining values were normalized to the total cell count per image (*Image_Count_Cells*) to calculate the percentage of each NETosis stage, which was then averaged across all fields of view (*Image_Metadata_Image*), and stratified by treatment (*Image_Metadata_Treatment*) and time point (*Image_Metadata_Hours*).

### Cross-dataset feature analysis and classifier performance

Manually annotated cell objects from all CPA training datasets (n=5 for HL-60 cells and n=4 for mouse bone marrow neutrophils), together with their corresponding CellProfiler-extracted features, were pooled to create combined datasets for downstream feature analysis. The HL-60 combined dataset comprised 4886 total cells (negative: 818 cells, spread: 735 cells, disintegrated nucleus: 763 cells, NETosis: 607 cells, dead: 1963), while the mouse neutrophil dataset included a total of 2183 cells (negative: 441 cells, spread: 496 cells, disintegrated nucleus: 364 cells, NETosis: 247 cells, dead: 635).

These combined datasets were used to train an independent Random Forest classifier implemented in Python (scikit-learn v1.3, Python v3.11) for feature interpretability and aggregate performance assessment. Random Forest classifiers were trained with 100 decision trees using stratified random sampling and a fixed random seed (16) to ensure reproducibility.

Feature importance scores were computed based on the mean reduction in Gini impurity for each feature across all trees in the Random Forest, resulting in ranked and normalized importance values. This analysis was performed to identify the most informative features that are consistently preserved across datasets, as feature importance and model structure cannot be readily extracted from CellProfiler Analyst. Univariate statistical comparisons of feature values were performed across all pairwise phenotype class combinations using the Mann-Whitney U test, with Holm correction applied to adjust p-values for multiple comparisons.

Classifier performance was evaluated using stratified 5-fold cross-validation, ensuring balanced representation of all five phenotypes within each fold. In each iteration, 80% of the data were used for training, while the remaining 20% were reserved as unseen data for testing. Performance metrics, including precision, recall and F1-scores for each class, were calculated using the function from scikit-learn, and results were averaged across all folds. Confusion matrices were computed for each fold, summed across folds, and row-normalized to visualize prediction outcomes and misclassifications for each true class. Importantly, all quantitative NETosis stage results reported elsewhere in the manuscript are derived from dataset-specific classifiers trained within CPA.

### Comparison of agreement between CPA trainers

Two independent users manually annotated cells from one of the HL-60 experimental datasets described above and trained separate CPA Random Forest classifiers to assess user-dependent variability in annotation and resulting cell counts. Training datasets were manually annotated into the five predefined NETosis stages, according to the previously described classification guidelines. A total of 1755 images (∼5.2 x 10^5^ cells) from all reported treatment conditions were included in this dataset and scored using the resulting classifiers.

Classification results were compared using Wilcoxon signed-rank tests with appropriate false discovery rate correction and rank-biserial effect sizes. Agreement and bias between classifiers were assessed and quantified using Spearman’s correlation coefficient (r) and ICC2 and ICC3 to evaluate inter-rater absolute agreement and consistency. Discrepancies between trainers were further characterized using mean absolute error (MAE) and Root Mean Square Error (RMSE).

### Comparison of NET quantification methods

To evaluate the agreement of CPA-derived NET counts generated from the two manual annotators (CPA count-trainer 1 and CPA count-trainer 2) with CellProfiler-derived counts (CP counts) and mean manual annotations, NET counts from 9 representative images (3 per treatment group) were compared using a non-parametric Friedman test with appropriate *post-hoc* tests and FDR correction.

Statistical comparisons were then repeated using the mean of CPA count-trainer 1 and CPA count-trainer 2. Rank-based agreement was quantified using Spearman’s correlation coefficient (r) to assess monotonic relationships between each pair of quantification methods, and Lin’s concordance correlation coefficients (CCC) were computed to evaluate overall agreement.

### Analysis of differences in NETosis dynamics

For each treatment condition, the median percentage of each cell class was calculated from the mean values per well. A mixed-effects model with independent experiments as a random intercept was used to account for per-experiment variability and *post-hoc* pairwise comparisons between relevant treatment groups were performed with FDR correction.

To quantify the cumulative magnitude of each phenotype over time, the temporal integral (TI) of each cell-state time course was calculated as the area under the curve using the trapezoidal rule across all replicates. Median TI values (expressed as % x hour) were computed for each treatment and NETosis stage to capture both the extent and duration of each phenotype. To assess treatment effects, a global linear mixed-effects model (LME) was fitted separately for each cell-state feature with Treatment as a fixed factor and Experiment as a random intercept, following the formula: TI ∼C(Treatment)+(1∣Experiment). Pairwise *post-hoc* comparisons between treatments were performed using reduced univariate LME models restricted to two treatments, with appropriate multiple testing correction. Median TI differences were reported to indicate the direction and magnitude of change between treatments.

The temporal composition of NETosis stages was analyzed using centered log-ratio (CLR) transformation to generate 3-component composition vectors (after adding pseudo-counts where necessary), where each vector represents the overall abundance and duration of the three NETosis cell phenotypes per replicate. Compositional differences between treatments were assessed using global and pairwise Permutational Multivariate Analysis of Variance (PERMANOVA, 999 permutations) based on Euclidean distances of CLR-transformed data, with p-values adjusted for multiple testing. The difference between treatment centroids in CLR space was calculated, and the relative contribution of each cell-state phenotype to the overall composition difference was determined as: %contribution=∑j​(ΔCLRj​)^2^/(ΔCLRi​)^2^​×100.

Key temporal metrics (Onset10, t_50_, and centroid) for each NETosis stage were derived from the time-course data using custom NumPy functions. To test for treatment effects on NETosis timing, LME models were fitted for each metric and cell feature, with treatment as fixed factor and experiment as a random intercept. We assessed monotonic trends across PMA doses, performed pairwise comparisons of PMA and ionomycin, and compared each stimulus with or without GSK484 pre-treatment. P-values were corrected for multiple comparisons.

### Implementation

All data included in this paper were handled using the pandas package (v2.2.2) in Python (v3.11). Data processing was performed using numpy (v1.26.4), scikit-bio (v0.6.3), and scikit-learn (v1.2.2). All statistical analyses were performed using scipy (v1.14.0), statsmodels (v0.14.2), and pingouin (v0.5.5), and visualizations were generated with matplotlib (v3.9.1) and seaborn (v0.12.2).

### Code availability

All analysis pipelines and classifier resources used in this study are publicly available. The full CellProfiler analysis pipeline and CPA classifier models for differentiated HL-60 cells and mouse bone marrow–derived neutrophils are available via GitHub at https://github.com/clandry091/NETosis-Stage-Classification-Pipeline.

## Results

### CellProfiler NETosis object segmentation and feature extraction

**Figure 2:**
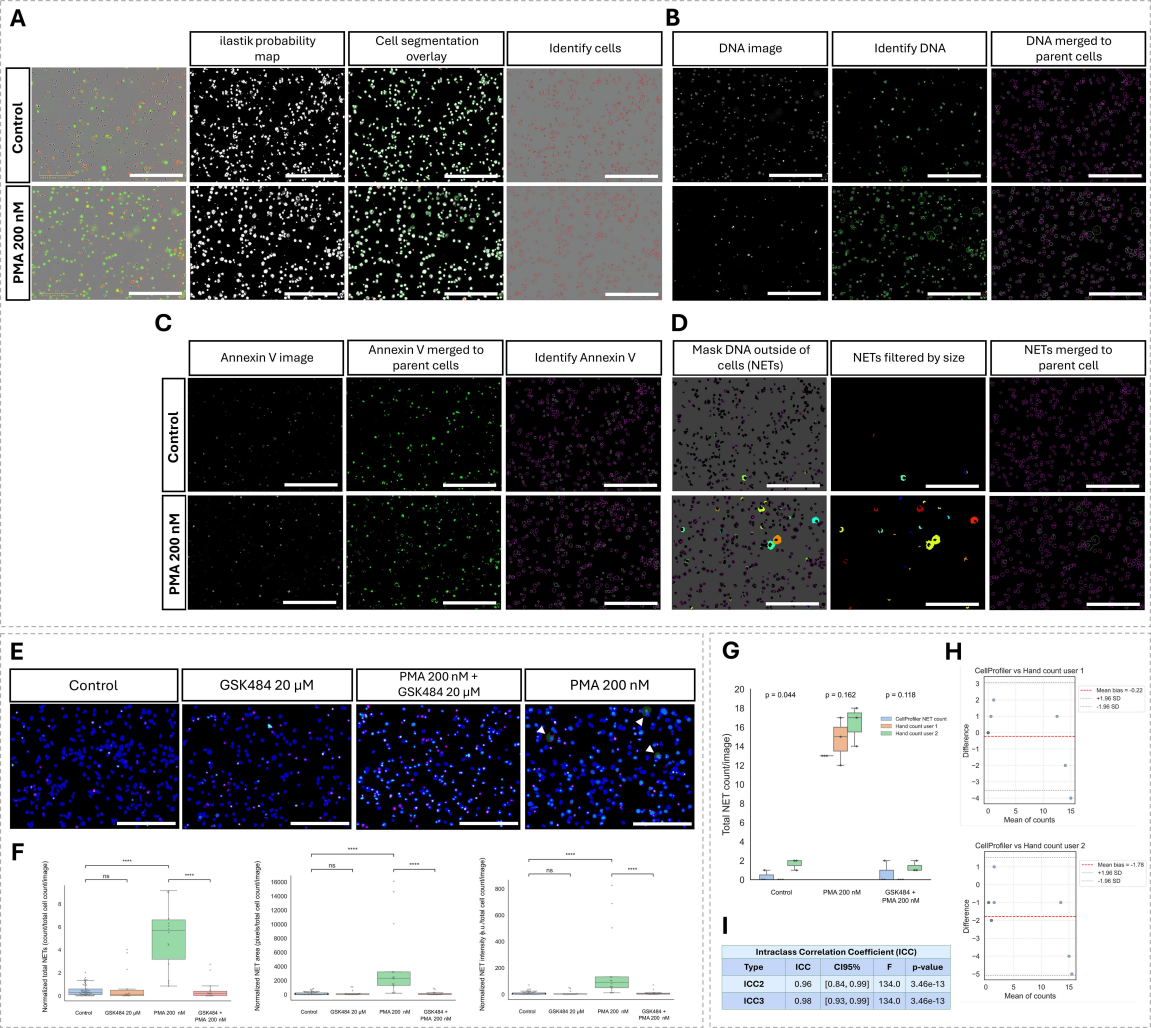
CellProfiler object-based segmentation and performance evaluation for NET detection in dHL-60s. **(A-D)**, Representative steps of the CellProfiler pipeline for segmenting cells, DNA, Annexin V and NETs, shown for untreated dHL-60 cells and those treated with 200 nM of PMA at 8 hours. Scale bar = 30 µm. **(A)**, ilastik-generated probability maps and corresponding CellProfiler segmentations overlaid on phase contrast images. **(B)**, Raw green fluorescence images (DNA) image, DNA object identification by CellProfiler and mapping to parent cells. **(C)**, Raw red fluorescence images (Annexin V), CellProfiler-identified Annexin V-positive regions, and their association with parent cells. **(D)**, NET segmentation from masking of DNA areas outside of segmented cells in CellProfiler and mapping to parent cells. **(E)**, Composite overlays showing segmented cells (blue), DNA (green) and Annexin V (red) with NETs outlined in red and indicated by the white arrows. Representative images are shown for control, 20 µM GSK484, 200 nM PMA, and GSK484 + PMA (30-min pre-treatment) conditions. Individual channel images corresponding to these examples are shown in Supplementary Fig. 2. **(F)**, Quantification of NET count, area, and fluorescence intensity per image across all four treatments. N=15, n=5. ****P<0.0001. **(G)**, Boxplot comparing NET counts from the CellProfiler pipeline with manual hand counts by two independent users. Kruskal-Wallis test results are shown above each treatment (N=3 per treatment). **(H)**, Bland-Altman plots illustrating the agreement and mean bias between the CellProfiler NET counts and each manual quantifier (N = 9 images). **(I)**, Intraclass Correlation Coefficients between CellProfiler NET counts and manual counts (N= 9 images).

The pipeline was initially developed and validated using neutrophil-directed HL-60 cells. Representative images illustrate the responsiveness of untreated cells and cells treated with 200 nM PMA, along with the subsequent image processing steps performed in CellProfiler (**Figures 2A–D**. Cell segmentation was performed on phase-contrast images using ilastik-generated probability maps. Resulting cell counts were comparable to manual annotations (**Supplementary Figure 1**), and segmented cell boundaries are outlined in red (**Figure 2A**). DNA objects were segmented from green fluorescence images and merged with their corresponding parent cells (**Figure 2B**). A similar process was applied to the red fluorescence images to identify and assign per-cell Annexin V objects (**Figure 2C**). NETs were then identified by segmenting extracellular DNA and applying a size threshold, followed by merging these objects to their respective parent cells (**Figure 2D**). PMA-treated cells show increased levels of DNA objects, many of which appear enlarged, reflecting elevated NET release (**Figure 2B**). While some level of spontaneous NETosis is observed in the control image, the representative PMA-treated image demonstrates a marked increase in both the number and size of segmented extracellular DNA structures (**Figure 2D**).

The pipeline was applied to quantify NET parameters in cells treated with 200 nM PMA and compared to untreated cells or those pre-treated with 20 μM GSK484 for 30 minutes prior PMA stimulation, to confirm that the pipeline accurately detected the expected reduction in NETosis with PAD4 inhibition. Representative overlay images generated by CellProfiler show clear NET release in PMA-treated cells, as in **Figures 2D**, whereas GSK484 pre-treatment shows complete suppression of extracellular DNA release, highlighting the sensitivity of the pipeline in detecting biologically relevant changes in NETosis (**Figure 2E**).

Total image-level NET features extracted from CellProfiler confirmed the expected effects of PMA and PAD4 inhibition (**Figure 2F**). PMA markedly increased NET count, area, and fluorescence intensity (all p<0.0001), whereas GSK484 pre-treatment alone did not induce NET release and reduced PMA-induced NETosis to near-baseline levels.

To assess the accuracy of the CellProfiler pipeline in identifying NETs, automated NET counts were compared to manual counts performed by two independent annotators across 3 representative images from the control, 200 nM PMA and GSK484 + PMA treatment groups. The pipeline demonstrated good overall agreement with manual counts. Although an overall difference was detected in control group, no pairwise comparisons reached statistical significance after correction, and the observed effect was largely attributable to variability between the two manual annotations (**Figure 2G**). Bland-Altman plots comparing CellProfiler-derived NET counts to those from manual annotations revealed a minimal bias toward underestimation by CellProfiler, a mean difference of -0.22 relative to Hand Count User 1 and -1.78 relative to Hand Count User 2 (**Figure 2H**), indicating strong concordance. Inter-rater variability between manual annotators was also assessed, showing a mean bias of -1.56 (**Supplementary Figure 3**), underscoring the potential for subjective variability and the value of automated pipelines in reducing user-dependent error.

To further evaluate the agreement between CellProfiler-generated NET counts and manual NET counts, ICCs were calculated (**Figure 2I**). The results demonstrated excellent concordance between methods. An ICC2 of 0.96 (p=3.46 x 10^-13^) indicates very strong agreement in absolute NET counts, while an ICC3 of 0.98 (p=3.46 x 10^-13^) reflects similarly high consistency in relative trends between methods.

### Evaluation of single-cell NETosis stage classification

**Figure 3:**
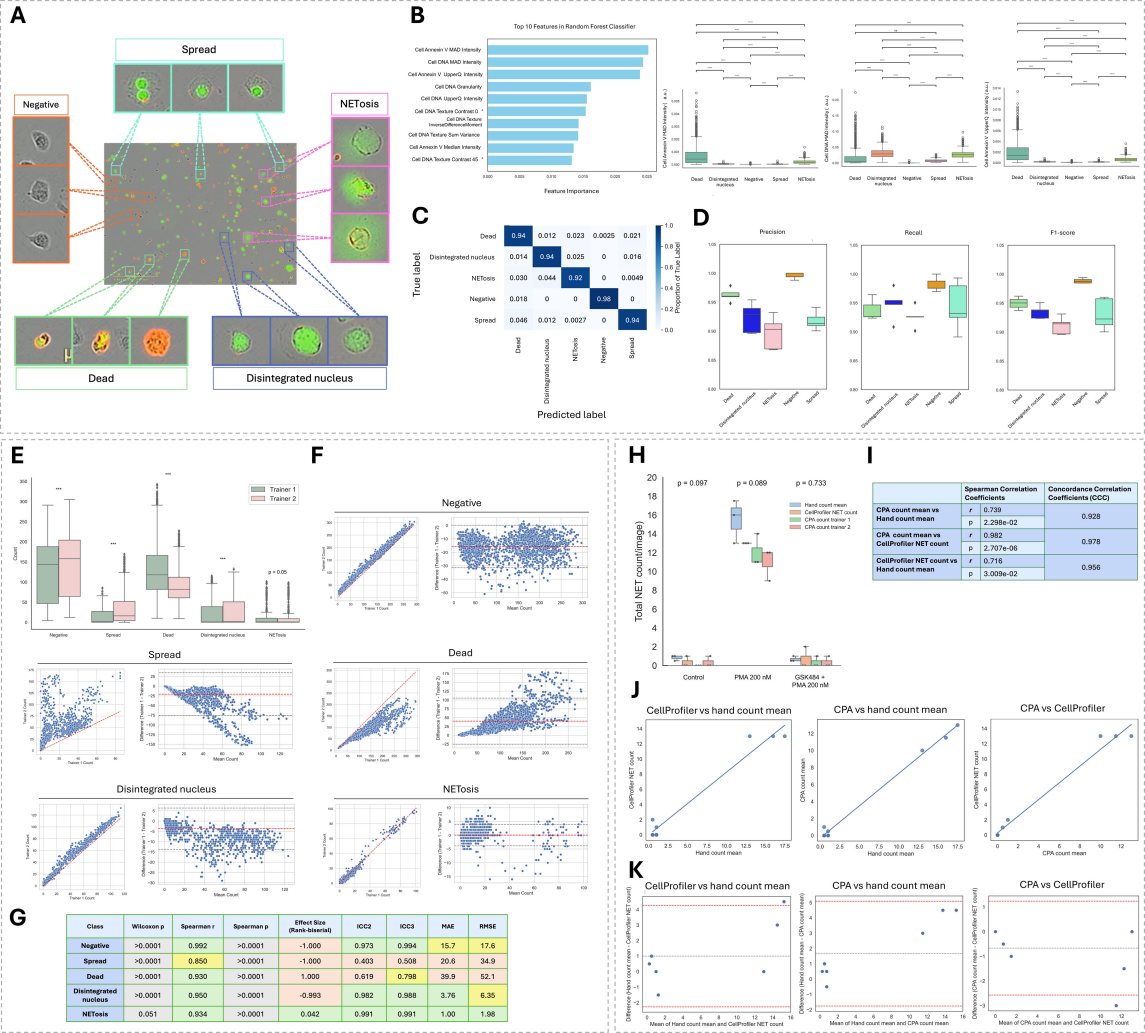
CellProfiler Analyst classification performance and inter-rater agreement across NETosis stages in dHL-60s. **(A)**, Representative images for each classification bin: Negative, Spread, Disintegrated nucleus, NETosis and Dead. **(B)**, Top 10 classification features in the Random Forest model ranked by importance. Boxplots on the right show the distribution of the top 3 features across classified cell types (n = 5 experiments, N = 4,886 cells) Mann-Whitney test and Holm correction performed. ****p<0.0001. **(C)**, Classifier Confusion Matrix on unseen, stratified test data from 5-fold cross validation (k=5). **(D)**, Classification performance metrics (precision, recall, and F1-score) for each cell type evaluated on stratified test data. **(E)**, Boxplots showing variability in predicted cell counts across two independently trained CPA classifiers. Paired Wilcoxon test with Benjamin-Hochberg correction performed. ***p<0.0001. **(F)**, Scatterplots and Bland-Altman plots showing agreement between two independently trained CPA classifiers for each NETosis stage. **(G)**, Summary of agreement metrics between the two CPA classifiers trained using independent annotation sets, including MAE, RMSE, Spearman ρ and p, ICC2, Wilcoxon p-values, and rank-biserial effect size. **(H)**, Boxplots comparing NET counts obtained from CellProfiler, two independently trained CPA classifiers, and the average of manual counts (n = 9 images). Statistical comparisons were performed using the Friedman test, with results annotated above each group. **(I)**, Summary of agreement between automated and manual NET counts assessed with Spearman correlation and Lin’s concordance correlation coefficients, n =9 images. **(J)**, Scatterplots comparing NET counts for each pairwise combination of NET quantification methods. **(K)**, Bland-Altman plots assessing agreement between each pair of quantifiers.

To evaluate single-cell NETosis stage classification performance, the Random Forest classifier for each experimental dataset was assessed for its ability to assign cells to five phenotypic categories: negative, spread, disintegrated nucleus, NETosis, and dead (**Figure 3A**). Classification accuracy for individual phenotypes varied between experiments, with overall classifier performance ranging from 90 to 95% accuracy (**Supplementary Figure 5A**). Spread and NETosis phenotypes showed greater variability, though still high-performing, with F1-scores ranging from 0.86 to 0.95 and 0.84 to 0.92, respectively (**Supplementary Figure 5B**). Training datasets from individual experiments were combined (N=4886 cells) to generate a single representative Random Forest Classifier tree (**Supplementary Figure 6**), allowing for evaluation of classifier performance across the full dataset and assessment of generalizability across experiments.

The classifier was characterized based on its top-ranking features across experiments to evaluate their contribution to phenotype discrimination (**Supplementary Figure 7**). Features extracted from phase-contrast images, DNA signal, and Annexin V signal within cell bodies were among the most informative (**Figure 3B**). Annexin V and DNA MAD intensities ranked the highest, with their upper quartile intensities also appearing among the top features. Additional important predictors included DNA granularity and several DNA texture features, such as contrast, inverse difference moment, and sum variance. These results highlight the value of DNA and Annexin V staining for distinguishing NETosis stages. The top three classification features, cell Annexin V MAD intensity, DNA MAD intensity and Annexin V upper-quartile intensity, differed significantly across phenotypes, except for DNA MAD intensity, which overlapped between dead and spreading cells (p=0.0746), making these two phenotypes more challenging to separate (**Figure 3B**).

To assess the power of these features and the overall performance of the Random Forest classifier in reliably sorting cells, classification accuracy was evaluated on unseen data using stratified test set evaluation. Classification metrics showed high accuracy in correctly identifying each phenotype. Cell labels were consistent with the training annotations 98% of the time for negative cells, 94% for dead, spread and disintegrated nucleus cells, and 92% for NETosis (**Figure 3C**). Accordingly, the median F1-scores followed a similar trend, with values highest to lowest for negative (0.988), dead (0.95), disintegrated nucleus (0.937), spread (0.923), and NETosis (0.914). Spread classification shows the greatest variability in F1-score, driven by inconsistent recall, suggesting variable sensitivity of the classifier in detecting this phenotype (**Figure 3D**). Precision was most variable for disintegrated nucleus and NETosis classifications, reflecting inconsistent rates of false positives across folds.

To evaluate the impact of inter-rater variability on the CPA training, classifier performance, and resulting cell phenotype counts, results obtained from two independent annotators using the same dataset were compared. A noticeable skew in results was observed across several phenotypes, driven by consistent user-specific biases during classifier training, which led to significant differences in the resulting cell counts. Specifically, counts of negative, spread, dead and disintegrated nucleus cells differed significantly, whereas NETosis counts did not (p=0.051) (**Figure 3E**).

Scatter plots comparing image-level cell counts revealed a strong correlation in NETosis counts (Spearman*r* = 0.934), with negligible inter-rater bias (effect size=0.042) (**Figures 3F, G**). Negative and disintegrated nucleus cells showed a modest yet consistent systemic bias, with slightly higher counts identified by Trainer 2’s classifier (negative: *r* = 0.992, effect size=-1; disintegrated nucleus: *r* = 0.95, effect size=-0.993). In contrast, spread and dead cells exhibited significant systematic deviations in opposite directions (spread:*r* = 0.85, effect size=-1; dead: *r* = 0.93, effect size=1), suggesting these phenotypes were interpreted differently by the two trainers and may have been inconsistently distinguished during classifier training.

Bland-Altman plots further supported these trends. NETosis counts showed a mean bias of only 0.04, reinforcing the consistency of classification for this phenotype. The mean differences in disintegrated nucleus (-3.73) and negative (-15.7) cell counts were marginal but consistent across the range of mean counts. Spread and dead cells displayed strong proportional bias, with differences increasing alongside the mean cell counts. Mean differences were −20.6 for spread and 39.9 for dead cells, indicating systematic under- and overestimation, respectively, between trainers.

To further quantify classifier agreement and prediction error, we evaluated absolute differences and intraclass correlation coefficients across phenotypes (**Figure 3G**). NETosis again showed the strongest agreement (ICC2/ICC3 = 0.991), with minimal prediction error (MAE = 1; RMSE = 1.98). Negative and disintegrated nucleus cells also demonstrated excellent agreement (ICC2 = 0.982 and 0.973, respectively) and moderate error rate (negative: MAE = 15.7, RMSE = 17.6; disintegrated nucleus= MAE = 3.76, RMSE = 6.35), consistent with modest inter-user bias. Dead cell classification showed moderate trend agreement (ICC3 = 0.798) yet displayed the highest prediction error (MAE = 39.9, RMSE = 52.1), indicating substantial inter-rater variability in labeling. Spread cells showed the lowest overall agreement (ICC2 = 0.403, ICC3 = 0.508) and high error rates (MAE = 20.6, RMSE = 34.9), suggesting that this phenotype is the most inconsistently interpreted and may benefit from clearer standardized annotation criteria to improve classification robustness.

To assess the accuracy of the CPA classifiers trained by two independent annotators in identifying NETs, automated NET counts were compared to those obtained from the CellProfiler segmentation pipeline and to the average manual counts across 3 representative images from the control, 200 nM PMA and GSK484 + PMA treatment groups, as shown in **Figure 3G**. Automated NET counts generated by the CPA classifier from trainer 1 and trainer 2 did not differ significantly from each other, from the hand count mean or CP counts across treatments (**Figure 3H**).

To evaluate the correlation of reported NET count across all methods, scatter plots were generated, and Spearman correlation coefficients calculated for each of the pairwise comparison of methods. The mean of CPA count-trainer 1 and CPA count-trainer 2 showed slightly stronger correlation with manual counts than those obtained directly from the CellProfiler pipeline (*r* = 0.74 vs *r* = 0.72) (**Figures 3J, I**). The highest correlation was observed between CellProfiler pipeline and CPA classifier outputs (r=0.98), showing consistency in automated methods.

Bland-Altman plots comparing mean differences in NET counts across each pairwise combination of methods revealed a modest bias toward underestimation by both automated methods relative to manual counts (CellProfiler=-1.667; CPA=-1). In contrast, the comparison between CellProfiler and CPA classifier average showed strong concordance, with a minimal mean bias of 0.667 (**Figure 3K**).

Overall agreement between methods, was excellent between CellProfiler and CPA (CCC = 0.978) and remained strong between CPA and manual counts, despite being the lowest among the comparisons (CCC = 0.928) (**Figure 3I**), highlighting that CPA classifiers are reliable when benchmarked against both manual and automated methods.

### Effect of pharmacological interventions on NETosis

**Figure 4:**
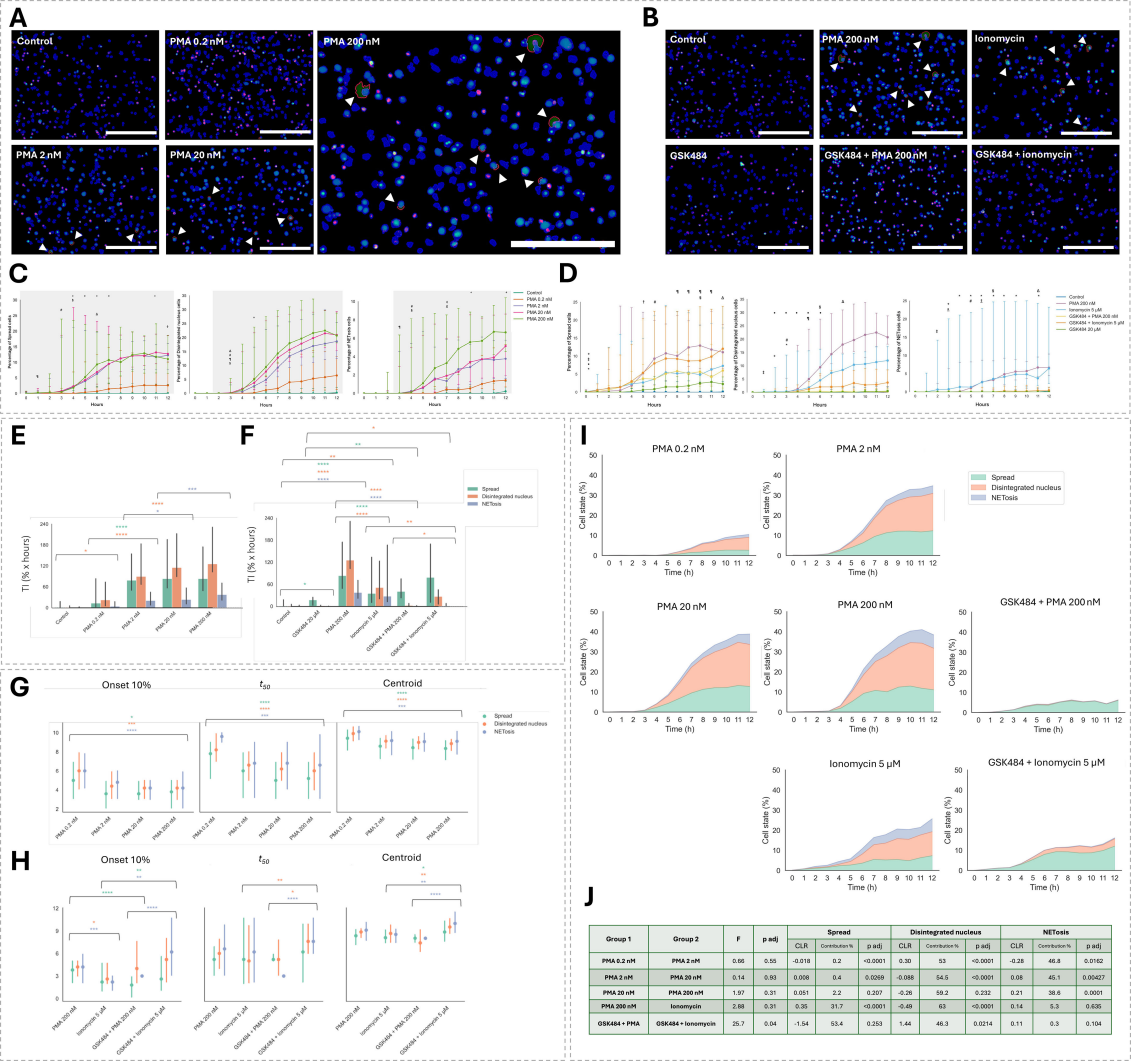
Assessment of changes in dHL-60 NETosis dynamics in response to distinct stimuli and PAD4 inhibition. **(A, B)**, Composite overlays showing segmented cells (blue), DNA (green) and Annexin V (red) with NETs outlined in red and indicated by white arrows. Representative images are shown for **(A)**, PMA curve (0.2-200 nM) and **(B)**, stimulation with 5 µM ionomycin or 200 nM PMA, with or without pre-treatment with GSK484 (30 minutes), after 8 hours. Individual channel images corresponding to these examples are shown in **Supplementary Figure 8**. **(C, D)**, Line plots showing the median percentage of each NETosis stage over time. Mixed-effects models were used with experiment as a random intercept, followed by Bonferroni-corrected pairwise comparisons. Symbols are shown only at the first significant time point if significance is maintained for all subsequent time points; otherwise, the symbol is shown at each significant time point. **(C)**, Increasing PMA concentrations. Grey boxes highlight time points where any PMA concentration is significantly higher than control. Pairwise comparisons are indicated as follows: ^§^ 0.2 vs 2 nM, ^¶^ 0.2 vs 20 nM, ^#^ 0.2 vs 200 nM, 2 vs 20 nM, Δ 2 vs 200 nM, ◊ 20 vs 200 nM (n = 5 experiments, N = 15 replicates). **(D)**, Pharmacological stimulation with ionomycin or PMA ± GSK484. Pairwise comparisons are indicated as follows: * Control vs GSK484 + Ionomycin, • Control vs GSK484 + PMA, ^†^ Control vs GSK484, ^‡^ Control vs Ionomycin, + Control vs PMA, Δ GSK484 + Ionomycin vs GSK484 + PMA, ^§^ GSK484 + Ionomycin vs Ionomycin, ^#^ GSK484 + PMA vs PMA, ^¶^ Ionomycin vs PMA (n = 5 experiments, N = 9 replicates). **(E, F)**, Temporal integral (TI) for each cell type with mixed-effects modeling with Holm step-down correction. n=5 experiments, N=15 replicates. *p<0.05, **p<0.01, ***p<0.001, ****p<0.0001. **(E)**, PMA curve (0.2-200 nM) and **(F)**, ionomycin and PMA with or without GSK484. **(G, H)**, Temporal metrics (0nset 10%, t50 and centroid) shown as medians with 95% confidence intervals. Differences between groups were tested using mixed-effect models with experiment used as a random intercept and Benjamini-Hochberg applied (n = 5 experiments, N = 15 replicates/treatment). **(G)**, Trend analysis across PMA concentrations (0.2-200 nM). **(H)**, Comparisons between PMA and ionomycin ± GSK484. **(I)**, Stacked area plot showing the composition of NETosis stages over time per treatment (n = 5 experiments, N = 15 replicates/treatment). **(J)**, Pairwise comparisons of NETosis stage composition across treatment conditions based on compositional log-ratio (CLR)-transformed data. For each comparison, the F statistic and Benjamini-Hochberg-adjusted p-values are reported. The table shows F-values and adjusted p-values (Benjamini–Hochberg correction) for each comparison, along with CLR differences and p-values per cell type. For NETosis, CLR-based TI is shown. Significant differences (*p < 0.05) are highlighted.

The assay was applied to characterize NETosis in neutrophil-differentiated HL-60 cells treated with increasing concentrations of PMA, allowing for evaluation of dose-dependent responses. NETosis induced by PMA was compared to that triggered by ionomycin (**Figure 4**). Both activators were assessed in the presence or absence of PAD4 inhibition with GSK484 to determine the contribution of PAD4 activity in each activation pathway. Data from five independent experiments were used, in which cells were stimulated with PMA at 0.2, 2, 20 or 200 nM or with 5 μM ionomycin, with or without a 30-minute pre-treatment with 20 μM GSK484 prior to stimulation with 5 μM ionomycin or 200 nM PMA. Across conditions, cells remained largely viable throughout the 12-hour treatment period, despite a gradual decline in viability over time (**Supplementary Figure 9**).

Representative overlay images show areas of NETs segmented by the CellProfiler pipeline at 8 hours post-treatment, delimited by red outlines. Cells exhibit increased NET formation in response to elevating PMA concentrations (**Figure 4A**). However, quantitative analysis of CellProfiler-extracted data revealed a significant increase in NET counts compared to control beginning at 2 nM PMA, with no discernible differences between higher concentrations (**Supplementary Figure 10A**). The absence of detection dose-dependent effects points to limited pipeline detection sensitivity or failure to capture earlier NETosis phenotypes which were detected when combined with the CPA classifier.

Overlay images from cells treated with 200 nM PMA or 5 μM ionomycin, with or without GSK484, display comparable levels of NETosis between both NET inducers, while GSK484 pre-treatment fully suppressed NETosis under both conditions (**Figure 4B**). Quantitative data extracted from CellProfiler corroborated these observations (**Supplementary Figure 10B**).

Temporal and compositional changes in NETosis dynamics were compared across treatments using the CPA classifier to quantify hourly levels of all three NETosis stages, enabling time-resolved comparisons between stimuli (**Figures 4C, D**).

Spreading induced by PMA significantly differed from untreated cells starting immediately after treatment onset. The three highest PMA doses (2, 20 and 200 nM) led to significantly more spreading than the lowest dose (0.2 nM), with divergence emerging around 1–4 hours post-treatment. Differences in spread cell percentages between the top three doses were minimal, indicating a similar degree of cell activation at doses above 2 nM (**Figure 4C**, panel 1).

Nuclear disintegration following PMA treatment became significantly different from control starting at 2 hours. By 3 hours, differences emerged between PMA doses, with all top three doses showing significantly higher disintegration than 0.2 nM. As the experiment progressed, some distinctions between the higher doses also appeared (at 5 hours: 2 vs 20 nM = 2.61% vs 3.75%, p=0.0005; 2 vs 200 nM = 2.61% vs 5.02%, p<0.0001). However, nuclear disintegration remained comparable between 20 and 200 nM PMA throughout (**Figure 4C**, panel 2).

NETosis became significantly elevated in PMA-treated groups compared to control starting at 3 hours, reflecting the staggered progression of NETosis stages, each separated by roughly 1 hour. Differences between PMA doses emerged around 3–4 hours, with the three highest doses inducing more NETosis than 0.2 nM. NETosis levels were comparable between 2 and 20 nM throughout most of the experiment, although 20 nM PMA produced higher NETosis by 12 hours (3.77% vs 5.15%, p<0.0001). In contrast, 200 nM induced significantly more NETosis than all other doses starting at 7 hours, reaching a median of 6.64% of cells in NETosis by 12 hours (**Figure 4C**, panel 3).

This time-course analysis revealed that while NETosis initiation is comparable in both timing and magnitude across PMA doses from 2 to 200 nM, higher doses lead to divergence at progressively later stages. Specifically, 20 nM induced more nuclear disintegration than 2 nM, whereas 200 nM surpasses 20 nM only at the stage of NET formation.

When looking at **Figure 4D**, all treatments led to elevated cell spreading compared to control, even in the presence of PAD4 inhibition. Stimulation with PMA or ionomycin, with or without GSK484 pre-treatment, significantly increased the proportion of spread cells starting at 1 hour post-treatment. GSK484 alone also induced cell activation, reflected by a significant increase in spread cells compared to control beginning at 5 hours (p=0.0021).

PAD4 inhibition with GSK484 dampened the magnitude of spreading in PMA-treated cells starting at hour 6 (PMA = 9.3% vs GSK484 + PMA = 3.61%, p=0.0033). In contrast, GSK484 pre-treatment followed by ionomycin stimulation had a synergistic effect, resulting in significantly more spread cells than ionomycin alone starting at 10 hours (ionomycin=4.82% vs GSK484 + ionomycin=8.74%, p= 0.022), and surpassing levels in the GSK+PMA group (at 12 hours: p=0.042).

Treatment with 200 nM PMA resulted in a faster accumulation of spread cells than ionomycin, with significantly higher levels observed between 8 and 11 hours post-treatment (at 10 hours: PMA = 12.94% vs ionomycin= 4.82%, p=0.00013).

An earlier onset of nuclear disintegration was observed with ionomycin, which significantly diverged from control starting at 1 hour post-treatment (p>0.0001), whereas this occurred at 2 hours with PMA. However, from 5 hours onward, PMA treatment led to higher levels of nuclear disintegration than ionomycin (at 5 hours: PMA = 5.02% vs ionomycin=1.77%, p=0.041).

PAD4 inhibition with GSK484 almost completely suppressed nuclear disintegration in PMA-treated cells, with significantly lower levels than PMA alone beginning at 3 hours (p=0.0252). Contrastingly, co-treatment with GSK484 and ionomycin still resulted in detectable nuclear disintegration, which was significantly higher than control starting at 3 hours (p=0.0094), though significantly reduced compared to ionomycin alone starting at 6 hours (ionomycin=4.5% vs GSK484 + ionomycin=2.03%, p=0.021). Accordingly, GSK484 + ionomycin led to a significantly greater proportion of cells with a disintegrated nucleus than GSK484 + PMA starting at 8 hours (GSK484 + ionomycin=2.10% vs GSK484 + PMA = 0.37%, p=0.0079).

In addition to an earlier onset of nuclear disintegration, ionomycin induced NETosis earlier than PMA, with significant increases compared to control observed at 2 hours (p=0.0058) and compared to PMA at 3 hours (p=0.0002). Despite this earlier onset, PMA and ionomycin resulted in similar levels of NETosis by 12 hours of stimulation. PAD4 inhibition with GSK484 significantly suppressed NETosis in both treatments, with significant divergence starting at 5 hours for PMA (p=0.00047) and at 7 hours for ionomycin (p=0.034). Although minimal, NETosis was still detected in ionomycin-treated cells despite PAD4 inhibition and was significantly higher than the GSK484 + PMA group (p<0.0001).

To assess differences in the magnitude of effect on each cell stage in response to increasing PMA concentrations, and under different stimuli with or without PAD4 inhibition, the TI was compared between relevant treatment groups (**Figures 4E, F**). Increasing concentrations of PMA led to progressive shifts in the proportion of cells in each phenotype (**Figure 4E**). The cumulative percentage of disintegrated nucleus cells increased significantly starting from the lowest concentration (0.2 nM) (Control=1.1 vs 0.2 nM=22.1, p=0.048), despite minimal and non-significant NETosis at this dose. The magnitude of nuclear disintegration (as measured by TI) continued to rise with increasing PMA concentrations, peaking at 20 nM and plateauing at 200 nM. The most substantial increase in cumulative cell spreading occurred between 0.2 and 2 nM (0.2 nM=12.88 vs 2 nM=78.42, p<0.0001), with levels remaining stable at higher concentrations, suggesting maximal cell activation from 2 nM. Cumulative NETosis began to significantly rise between 2 and 20 nM (p=0.013), with a pronounced increase observed at 200 nM (p=0.00014).

Treatment with 200 nM PMA significantly increased the magnitude of induction across all NETosis stages compared to control (**Figure 4F**). In contrast, ionomycin elicited variable responses between experiments, only consistently increasing the proportion of disintegrated nucleus cells over time. When comparing the two stimuli, PMA induced greater levels of both spread and disintegrated nucleus phenotypes than ionomycin (both p<0.0001), while NETosis was comparable between treatments.

The impact of pre-treatment with GSK484 on the overall levels of each cell type with each NET inducer was assessed. Interestingly, GSK484 alone led to an increase in cell spreading. When combined with PMA, this effect was further amplified (17.1 vs 40.18, p=0.0066). However, PAD4 inhibition suppressed the induction of disintegrated nucleus cells and NETosis triggered by PMA. With ionomycin, GSK484 co-treatment significantly reduced the proportion of disintegrated nucleus cells to 26.64, although levels remained higher than with GSK484 alone (17.1, p=0.012). While NETosis levels were nearly absent in the GSK484 + ionomycin condition, variability between replicates prevented this reduction from reaching statistical significance when compared to ionomycin alone. Notably, GSK484 + ionomycin resulted in a greater accumulation of disintegrated nucleus cells than GSK484 + PMA (26.64 vs 2.09, p=0.048), suggesting stimulus-specific differences in PAD4-dependent regulation of NETosis.

To assess temporal dynamics in NETosis progression across varying doses and stimuli, timing metrics for each cell type were compared between treatment conditions (**Figures 4G, H**). Specifically, we tested for dose-dependent monotonic trends in the onset, T_50_ and centroid with increasing PMA concentrations (**Figure 4G**). The initiation, rate of progression, and timing of peak response for each NETosis stage increased in a dose-dependent manner. At 0.2 nM, the median onset times for cell spreading, nuclear disintegration and NETosis were 5, 6, and 6 hours, respectively, while at 200 nM, these values shifted to 4, 5, and 5 hours. Higher PMA concentrations led to faster progression across all cell types, with cell spreading, nuclear disintegration and NETosis reaching 50% of their maximum value 3, 1, and 2 hours earlier, respectively, at 200 nM) compared to 0.2 nM. The overall temporal distribution of the response for each cell type, as measured by the centroid, closely mirrored the T_50_ trend, occurring progressively earlier with increasing PMA concentration (all p<0.001), indicating a globally accelerated NETosis response with higher stimulation levels.

When comparing the temporal dynamics of PMA and ionomycin stimulation, ionomycin induced an earlier onset of nuclear disintegration (p=0.013) and NETosis (p=0.0004), while the subsequent progression rate, as measured by the T_50_ and centroid values, was comparable between the two stimuli. With GSK484 pre-treatment, PMA-induced cell spreading occurred earlier compared to PMA alone (p>0.0001). However, no significant differences were observed in the timing of nuclear disintegration and NETosis under PAD4 inhibition with PMA, which is expected given the near absence of these cell types in the GSK484 + PMA group. In contrast, co-treatment with GSK484 and ionomycin delayed the onset of nuclear disintegration (p=0.009) and NETosis (p=0.0061) relative to ionomycin alone and slowed the progression of disintegrated nucleus cells progression to 50% of their maximum value (p=0.0062). Overall, GSK484 attenuated the response to ionomycin across all three phenotypes, as indicated by higher centroid values compared to ionomycin alone. While temporal comparisons between GSK484 + PMA and GSK484 + ionomycin were conducted, the absence of nuclear disintegration and NETosis in the GSK484 + PMA condition limits the relevance of these results.

To assess compositional differences in the distribution of the three cell phenotypes, stacked area plots were generated to visualize treatment-specific shifts in relative abundance (**Figure 4I**). Relative abundances were transformed using the CLR to test for proportional changes between treatments (**Figure 4J**). A significant difference in overall cell composition was observed between PMA- and ionomycin-stimulated cells in the presence of PAD4 inhibition (p=0.04). In contrast, no significant differences were detected in the distribution of NETosis stages across increasing PMA concentrations or between PMA and ionomycin stimulation alone.

Although global compositional differences between increasing concentrations of PMA were not statistically significant, visual inspection of stacked area plots and pairwise comparisons revealed dose-dependent shifts in the distribution of cell phenotypes. Up to 20 nM, sequential increases in PMA concentration led to shifts in all three phenotypes, although disintegrated nucleus cells and NETosis accounted for ~50% and ~40% of the observed difference, respectively. Between 20 nM and 200 nM, compositional differences were predominantly driven by an increase in NETosis (>30%, p=0.0001). Although disintegrated nucleus cells contributed 59.2% of the overall shift, their relative abundance did not differ significantly between the two groups due to high inter-replicate variability (p=0.232).

When comparing PMA 200 nM to ionomycin, ionomycin stimulation resulted in a lower abundance of spread cells and a higher abundance of disintegrated nucleus cells compared to PMA 200 nM, accounting for 31.7% and 63% of the observed composition difference, respectively, while NETosis proportion remained similar. Under PAD4 inhibition, 46.3% of the difference in composition between PMA and ionomycin treatments was attributable to a higher abundance of disintegrated nucleus cells in the ionomycin group (p=0.0214). Although spread cells also contributed to the shift, the difference was not statistically significant.

Together, these analyses of treatment-specific NETosis dynamics revealed progressive phenotype-specific increases in both the rate and magnitude of NETosis stages in response to sequential increases in PMA concentrations, with NETosis abundance primarily driving the shift between the highest concentrations. Ionomycin was observed to induce earlier onset of NETosis than PMA, although overall NET release remained similar. Results also revealed differential responses to PAD4 inhibition between the two stimuli, showing a reduced yet sustained capacity for nuclear disintegration with ionomycin stimulation, whereas this was markedly reduced with PMA. These findings suggest that PMA and ionomycin activate distinct pathways and that nuclear disintegration is not strictly PAD4-dependent under ionomycin stimulation.

### NET assay validation in WT and *Padi4*^-/-^ mouse neutrophils

**Figure 5:**
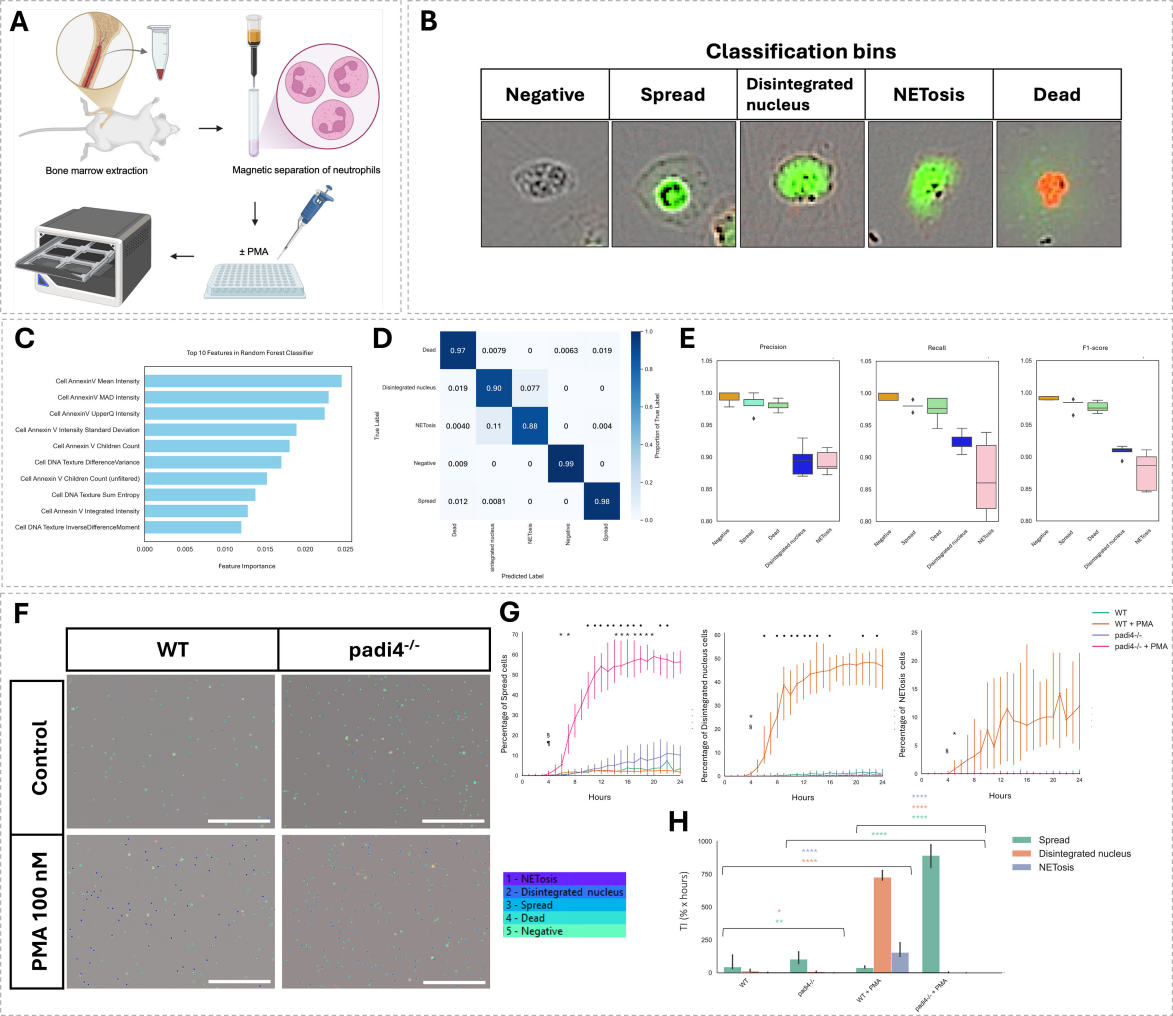
CellProfiler Analyst classification performance and NETosis dynamics in mouse bone marrow neutrophils. **(A)**, Schematic of the workflow for magnetic separation of bone marrow-derived neutrophils and their application in the NET assay. Created in BioRender. **(B)**, Representative images for each classification bin: Negative, Spread, Disintegrated nucleus, NETosis and Dead. **(C)**, Top 10 classification features ranked by importance in the Random Forest model (n = 4 experiments, N = 2183 cells). **(D)**, Confusion Matrix of classifier predictions on unseen, stratified test data from 5-fold cross validation (k=5). **(E)**, Classification performance metrics (precision, recall, and F1-score) for each phenotype, evaluated on stratified test data. **(F)**, Composite overlays with distinct colors indicating predicted class labels for each cell. Representative images are shown for neutrophils from WT and *Padi4*^-/-^ after 8 hours, with or without stimulation with 100 nM PMA. Scale bar=300 µM. Individual channel images corresponding to these examples are shown in **Supplementary Fig. 14**. **(G)**, Line plots showing the median percentage of each NETosis stage over time. Mixed-effects models with experiment as a random intercept were followed by Bonferroni-corrected pairwise comparisons. Symbols are shown only at the first significant time point if significance is maintained for all subsequent time points; otherwise, symbols mark each significant time point. Pairwise comparisons: * WT vs WT + PMA, • WT vs *Padi4*^-/-^, § WT + PMA vs *Padi4*^-/-^ + PMA, ¶ *Padi4*^-/-^ vs *Padi4*^-/-^ + PMA. (n = 4 experiments, N = 12 replicates). (H), Temporal integral (TI) for each NETosis phenotype in WT and *Padi4*^-/-^ neutrophils with or without PMA. Mixed-effects modeling with Holm step-down correction was applied. n=4 experiments, N=12 replicates. *p<0.05, **p<0.01, ****p<0.0001.

The assay was applied to characterize NETosis in mouse bone marrow-derived neutrophils harvested from WT and *Padi4*^-/-^ mice, allowing evaluation of its accuracy in confirming the lack of NET formation in *Padi4*^-/-^ cells and assessing the impact of PAD4 deficiency on NETosis progression. Bone marrow was harvested from one femur of WT and *Padi4*^-/-^ mice by centrifugation, and neutrophils were isolated using magnetic separation (**Figure 5A**). Cells were either left untreated or stimulated with 100 nM PMA. Following feature extraction in CellProfiler (**Supplementary Figure 11**), cells were classified into predefined categories: negative, spread, disintegrated nucleus, NETosis, and dead for single-cell classification (**Figure 5B**).

CPA classification showed consistently high performance across four independent datasets, with strong discrimination between NETosis and non-NET phenotypes (**Supplementary Figure 12**). The classifier was characterized based on its top-ranking features across experiments to evaluate their contribution to distinguishing between cell phenotypes (**Supplementary Figure 13**). Annexin V signal intensity and children count within cells were among the most important discriminators, along with DNA texture features (**Figure 5C**). Annexin V mean intensity, MAD intensity, and upper quartile intensity ranked highest. For the DNA signal, texture difference variance was ranked as the most influential feature, followed by texture sum entropy, and inverse difference moment.

The overall performance of the Random Forest classifier in accurately sorting cells was evaluated using stratified test set validation on unseen data. Mean classification accuracy for each phenotype is summarized in a confusion matrix (**Figure 5D**), with cell labels matching the training annotations 97% of the time for dead cells, 90% for disintegrated nucleus, 88% for NETosis, 99% for negative cells, and 98% for spread cells. Misclassification was most frequent between disintegrated nucleus and NETotic cells, with 7.7% and 11% of each phenotype misclassified as the other.

Consistent with this, NETosis displayed the lowest and most variable median F1-score (0.887), largely due to variable recall, followed by disintegrated nucleus (0.91) (**Figure 5E**). Negative, spread, and dead cells were detected with high accuracy and precision, reflected in median F1-scores of 0.994, 0.985, and 0.976, respectively.

Representative classified images show predominantly disintegrated nucleus and NETotic cells in WT neutrophils treated with PMA, whereas the same treatment in *Padi4*^-/-^ neutrophils resulted mainly in an increase in spread cells (**Figure 5F**).

Time-course analysis of the median percentage of spread cells revealed that this phenotype appeared earliest in PMA-stimulated *Padi4*^-/-^ neutrophils and was significantly higher than in both untreated *Padi4*^-/-^ neutrophils (p=0.0045) and PMA-stimulated WT neutrophils (p=0.002) from 4 hours onward, reaching a maximal response at 20 hours with a median 59% of spread cells (**Figure 5G**). Accordingly, the median TI for spread cells in this group showed approximately a 9- and 23-fold increase compared with the other two conditions (**Figure 5H**).

Spread cells were also detected in unstimulated *Padi4*^-/-^ neutrophils at levels significantly higher than those in WT neutrophils, regardless of PMA treatment, with a maximal value of 11% at 22 hours. In WT neutrophils, PMA stimulation triggered a faster rise in spread cells around 5–6 hours (p=0.0014). However, levels in untreated WT neutrophils eventually surpassed those in PMA-treated WT cells by 21 hours (p=0.0042), although the overall magnitude of spread cells, as reflected by the TI values, did not differ significantly between these two groups (**Figure 5H**).

This pattern likely reflects progression of PMA-treated WT neutrophils to later NETosis stages. Consistent with this, disintegrated nucleus cells increased markedly from 4 hours in PMA-stimulated WT neutrophils, reaching significantly higher levels than in untreated WT (p=0.0002) and PMA-stimulated *Padi4*^-/-^ neutrophils (p<0.0001) starting at 4 hours. PMA-treated WT neutrophils reached a maximum of 48.4% at 22 hours. *Padi4*^-/-^ neutrophils, whether treated or untreated, showed no disintegrated nucleus cells. Untreated WT neutrophils showed only minimal levels of this phenotype, although still significantly higher than in *Padi4*^-/-^ neutrophils at several time points after 6 hours. These differences were also reflected in the corresponding TI values (**Figure 5H**).

Only PMA-stimulated WT neutrophils progressed to a more advanced NETosis phenotype, representing approximately 12% of cells at 24 hours, and becoming significantly higher than in untreated WT neutrophils (p=0.0068) and PMA-stimulated *Padi4*^-/-^ neutrophils (p=0.0268) starting at 5 and 4 hours, respectively, with these differences mirrored in the TI results (**Figure 5H**). Notably, unstimulated WT neutrophils exhibited negligible terminal NETosis. While literature reports suggest spontaneous NETosis in 8–10% of unstimulated peripheral blood neutrophils (26), our results may be reflective of the negative isolation method, which avoids mechanical priming (27, 28), and the inherently quiescent state of bone marrow neutrophils (29). Our data suggest these cells instead default to constitutive apoptosis (30) (**Supplementary Figure 15**).

Together, these analyses demonstrate that the NET assay and analysis pipeline can reliably distinguish between NETosis stages in mouse bone marrow-derived neutrophils, with minimal misclassification between disintegrated nucleus and NETotic cells. Consistent with the essential role of PAD4 in NET formation (20), the pipeline accurately captures the absence of NET formation in *Padi4*^-/-^ neutrophils, which showed only accumulation of spread cells following PMA stimulation, while detecting late-stage NETosis exclusively in PMA-treated WT neutrophils, highlighting the assay’s ability to resolve PAD4-dependent differences in NET formation.

## Discussion

Here we present a high-throughput methodology and analysis pipeline for tracking of NET release across its various morphological stages (14, 15, 31), enabling inference of underlying mechanisms and progression dynamics. By segmenting and characterizing single-cell features, this comprehensive approach identifies distinguishing traits of each NETosis stage and supports the development of robust algorithms for automated single-cell classification. The live-cell imaging assay concurrently captures the timing and progression of NETosis under diverse conditions, providing quantitative insights into the magnitude of responses, the sequence of morphological transitions, and the composition of cell populations, and the mechanisms governing temporal dynamics.

The live-cell imaging assay offers distinct advantages over conventional immunofluorescence staining protocols, eliminating the need for extensive sample manipulation and fixation that can alter NET structures and limit temporal resolution (32). Fixation causes NETs to collapse and spread across the field of view, leading to extensive overlap that precludes direct application of the current pipeline to fixed samples. The 96-well format described here requires few cells and minimizes sample preparation time, making it well-suited for high-throughput screening applications.

Notably, end-point only measurements of NET release can fail to capture stimulus-dependent differences in NETosis (33), underscoring the importance of defining temporal dynamics. Our analysis pipeline performs automated quantification of conventional NET metrics over time, including NET count, area and fluorescence intensity, thereby reducing the well-recognized potential for user bias (32). Beyond standard quantification, our pipeline integrates morphological features from phase-contrast images, a source of valuable mechanistic insights (15). By leveraging the ilastik Random Forest classifier in combination with CellProfiler, we achieved improved segmentation of low-contrast cells. Together with DNA and Annexin V staining data, these image features enable characterization of sequential cellular changes and distinction between apoptotic and NETotic cells, a well-recognized pitfall in NET research (15, 32). These two pathways of neutrophil cell death exhibit differing morphological features, membrane integrity profiles, and Annexin V staining (15, 31), which are more easily discernable with this imaging methodology. The comprehensive feature characterization, coupled with CPA, also enables the identification of key NET features that capture the full spectrum of NET phenotypes (6, 7, 14), from onset to release. This enhances assay sensitivity and provides a more accurate and comprehensive view of NETosis, enabling detection of subtle dose- and stimulus-dependent differences.

Comprehensive single-cell characterization further supports the adaptability of the pipeline for neutrophils from various sources. Notably, human and mouse neutrophils have been reported to exhibit distinct NETting patterns (34), highlighting the need for an adaptable, non-stringent analysis workflow. Such flexibility is supported by a Random Forest framework such as the one implemented in CPA, which classifies cells using a fixed set of CellProfiler-derived measurements while allowing classification rules to adapt across experiments and accommodate heterogeneous, overlapping phenotypes within predefined bins (35). The compatibility of CellProfiler and CPA enables consideration of all extracted features without bias (19), facilitating the development of classification algorithms that preserve this adaptability. Lastly, this approach generates sufficient single-cell data to resolve key steps preceding NET release, offering valuable insight for inferring NETosis induction mechanisms, defining activation timing, and pinpointing the stages at which various mediators contribute to the process.

Sequential stages of neutrophil activation and NET release have been described previously, categorized as phase 1 (P1), phase 2 (P2), and phase 3 (P3) in one study (31), and were primarily defined based on similar morphological changes (14, 15, 31). In human blood neutrophils, PMA stimulation induces cell body spreading as early as 30 minutes, followed by nuclear swelling that fills up the intracellular space in most cells between 80–120 minutes, with NETosis reported between 120–220 minutes. In contrast, our results show a later onset of each stage in neutrophil-directed HL-60s and in mouse bone marrow neutrophils, which have been reported to release fewer NETs and form less prominent network structures (34). Despite these temporal differences, HL-60 cells progressed through analogous morphological stages to those described in primary neutrophils, supporting the utility of this system for method development and high-throughput screening. Accordingly, our approach captured the previously reported differential progression of NETosis between PMA and ionomycin, identifying an earlier onset of cell activation and NETosis with ionomycin (33).

Endpoint NET measures remain a valuable tool, having revealed, for example, a PMA dose-dependent increase in NET levels (36, 37). Our methodology extends these findings by revealing the altered dynamics underlying differences between concentrations. Similarly, endpoint analyses have shown that PAD4 inhibition abrogates NET release in response to both PMA and ionomycin (38). While our results are consistent with this, temporal analysis revealed stimulus-specific effects of PAD4 inhibition and a greater propensity for cell spreading in *Padi4*^-/-^ mouse neutrophils, consistent with reports that these cells generate higher levels of reactive oxygen species (ROS) and display enhanced activation (38). Together, these observations highlight that commonly used fixed endpoints (4–6 hours) may not fully capture the extent of NETosis responses, which vary in both timing and magnitude across stimuli and cellular sources. Resolving temporal dynamics therefore enables more accurate comparison of NETosis across experimental systems.

A key strength of our methodology is its versatility for mechanistic studies of NETosis. When paired with pharmacological interventions targeting specific NET mediators, it can be used to elucidate mechanisms of NETosis inhibition, degradation, and regulation. Its adaptable design allows tracking of single-cell progression through NETosis stages (*TrackObjects* in CellProfiler), enabling detailed examination of associations and transitions between stages. The pipeline is also flexible in its staining requirements. Although Annexin V staining provides valuable information for distinguishing NETosis stages, we achieved approximately 88% classification accuracy with DNA staining alone. This allows Annexin V to be replaced with fluorescent labeling of alternative targets for added mechanistic insight, although it may impact classification performance.

The staining protocol described here primarily supports visual classification of the most prominent DNA-associated NETosis stages, with morphological information provided by the phase-contrast images. Intermediate steps of NETosis, such as actin remodeling and microvesiculation, have been described and may be partially discernable in phase-contrast images (13). However, the current protocol does not provide sufficient information to reliably annotate these features during classifier training. Capturing these processes would likely require changes to the staining strategy or the use of higher-magnification imaging.

This also applies to plasma membrane rupture, which has been classified by other groups as a distinct stage preceding NET release (13, 16). In the present pipeline, the onset of membrane permeabilization is inherently detected, as the membrane-impermeable dye becomes visible upon initial loss of membrane integrity prior to extracellular DNA release. However, intermediate permeabilized states are not defined as standalone classes until DNA release is observed, marking membrane rupture. Differences from other classification frameworks therefore reflect differences in staging granularity and would require adaptation of staining strategy to investigate.

It is also important to note that the present pipeline categorizes terminal NETosis solely by the release of extracellular DNA. While this does not capture all molecular features traditionally used to define NETosis (39), it enables high-throughput characterization of NET release dynamics suitable for screening applications. Accordingly, this approach is intended to complement established NET detection methods and should be paired with immunological staining to confirm the presence of canonical NET-associated markers.

As an additional specificity control, pairing treatments with a NETosis inhibitor such as GSK484 can help distinguish NET-associated DNA release from signals arising from other forms of cell death in the absence of molecular NET markers. This approach enabled the identification of an “unexpected non-viable cell phenotype” in bone marrow neutrophils, observed across all conditions. We tracked their temporal behavior and found that they originated as small, non-adherent cells exhibiting DNA-positive staining at time 0. Together, these features suggest that they represent necrotic cells that progressively lose membrane integrity over time (40), resulting in the appearance of a NET-like extracellular DNA cloud independent of experimental stimulation.

In addition to classification scope, several technical and biological factors contribute to variability in NETosis measurements. Although the mean proportions of each stage are consistent between cell populations, individual cells display a wide range of onset times for the different phases (14, 31), introducing variability between experiments. Consequently, large sample sizes are required to reliably detect differences and achieve adequate statistical power.

Brightfield images are more challenging to segment than fluorescence images due to overlapping intensity values between cells and background. While classical filters can be applied, they are sensitive to morphological variability and often require expert tuning. In contrast, ilastik-generated probability maps accommodate image variability through user-provided training labels and offer a shorter learning curve for new users. However, the low contrast of activated and NETting cells in phase-contrast images can still lead to segmentation errors in ilastik, including over-segmentation of single cells despite thorough annotation.

In addition, although the general CPA model architecture can be reused, classifiers must be retrained for each individual dataset to account for differences in imaging conditions and cell populations. While this introduces an additional annotation requirement, retraining can be guided by predictions from a previously trained classifier, enabling rapid selection of correctly assigned cells and experiment-specific refinement rather than extensive manual re-annotation. However, differences in user-generated CPA training datasets may introduce systematic bias in classification, particularly for phenotypes that are less sharply defined morphologically. Notably, the relatively low inter-rater agreement for the spread phenotype may reflect biological heterogeneity within this intermediate state, consistent with prior reports suggesting the presence of multiple subpopulations among cells that share the molecular and morphological features used here to define the spread phenotype (16). Although NETting cell quantification was consistent between users, this bias should be considered when interpreting the data, with greater emphasis placed on trends rather than absolute cell counts for other stages.

Finally, the pipeline provides only 2D resolution, which limits the complete detection of NETs and accurate reporting of their area. Moreover, nuclear swelling prior to NET release has been associated with a gradual increase in cell height (14, 31), a parameter not captured by the current analysis pipeline and therefore excluded as a metric, which can hinder distinction from NETting cells. Despite these limitations, the ability of our assay to monitor NETosis at single-cell resolution and across defined morphological stages provides a powerful tool for addressing outstanding questions in the field.

## Data Availability

The raw data supporting the conclusions of this article will be made available by the authors, without undue reservation.
