# Supplementary material for: A semi-automated imaging and analysis pipeline for NET quantification and temporal-profiling of NETosis
Source: Front Immunol. 2026 Mar 11;17:1753477. doi: 10.3389/fimmu.2026.1753477 (PMC13012954; doi:10.3389/fimmu.2026.1753477)
Supplement: Supplementary file 1 [file DataSheet1.pdf]

Supplementary Methods S1 : Regular expression used for metadata extraction

The following regular expression was used in CellProfiler to extract metadata fields (plate, well, image number, day, hour, and minute) from Incucyte image file names:

```
^(?P<Plate>.*)_(?P<Well>[A-P][0-9]{1,2})_(?P<Image>[0-9])_(?P<day>[0-9]{2})d(?P<hours>[0-9]{2})h(?P<minute>[0-9]{2})m
```
