# Supplementary material for: A semi-automated imaging and analysis pipeline for NET quantification and temporal-profiling of NETosis
Source: Front Immunol. 2026 Mar 11;17:1753477. doi: 10.3389/fimmu.2026.1753477 (PMC13012954; doi:10.3389/fimmu.2026.1753477)
Supplement: Supplementary file 2 [file Presentation1.pptx]

## Slide 1
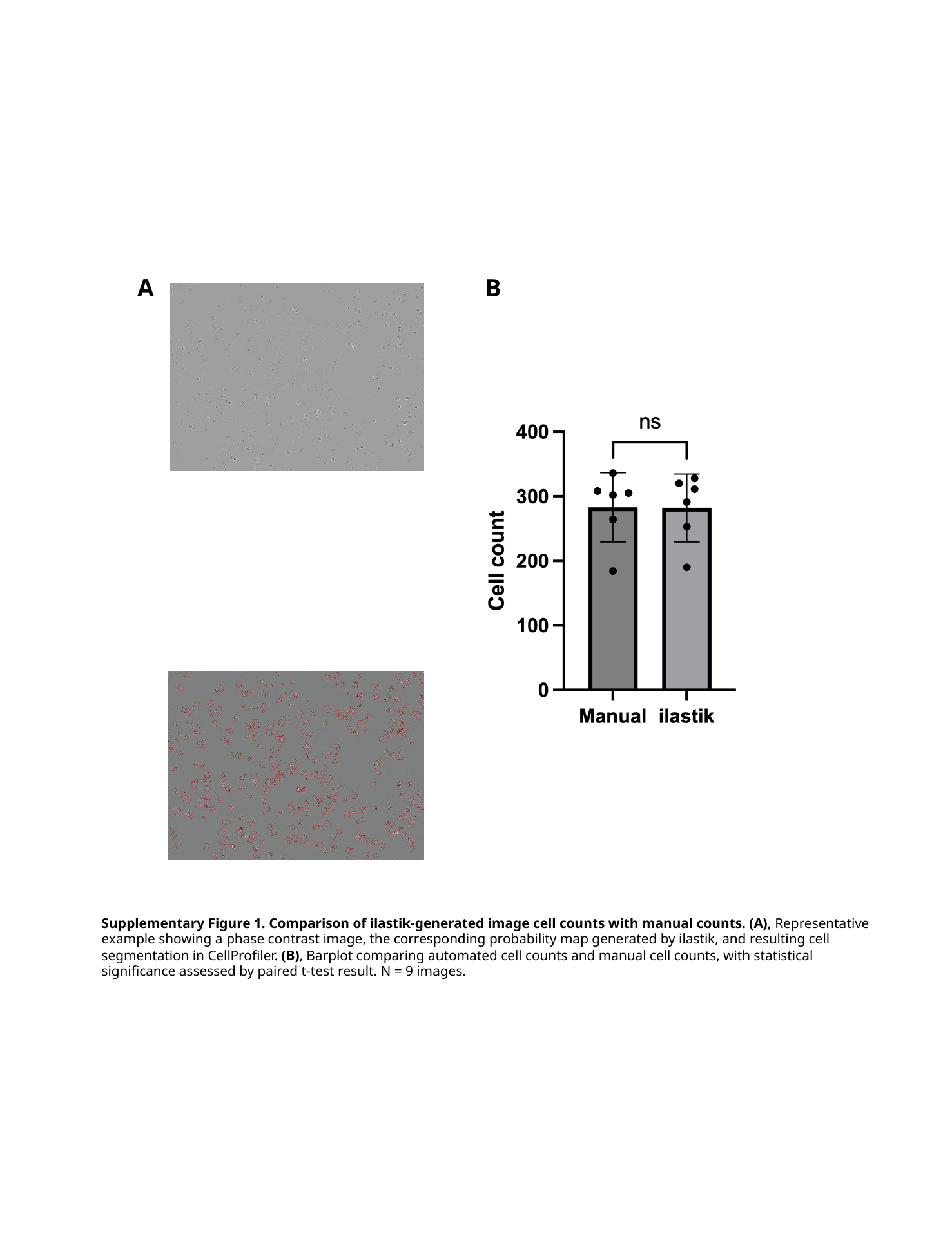

A
B
Supplementary Figure 1. Comparison of ilastik-generated image cell counts with manual counts. (A), Representative example showing a phase contrast image, the corresponding probability map generated by ilastik, and resulting cell segmentation in CellProfiler. (B), Barplot comparing automated cell counts and manual cell counts, with statistical significance assessed by paired t-test result. N = 9 images.

## Slide 2
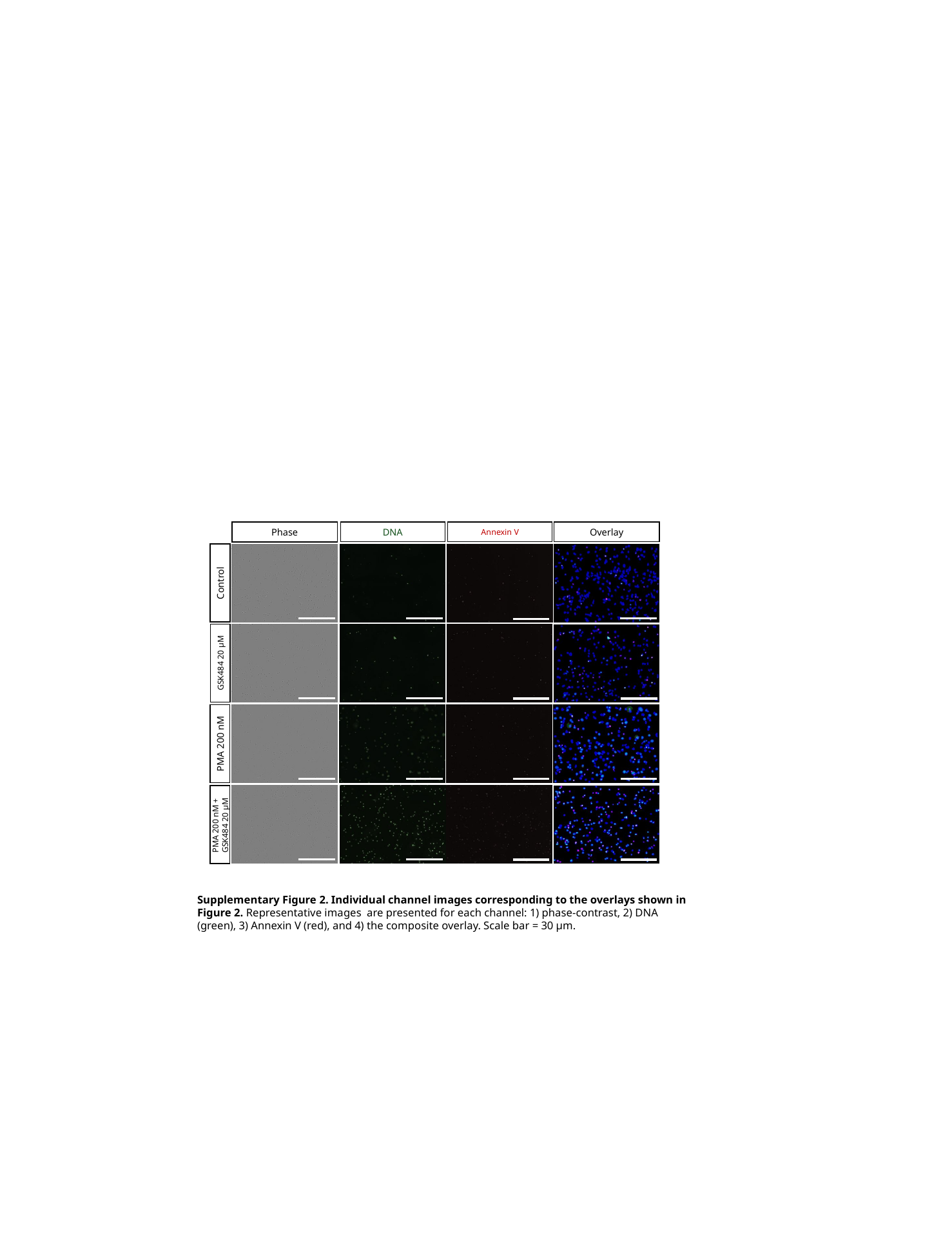

Annexin V
Phase
DNA
Overlay
Control
GSK484 20 µM
PMA 200 nM
PMA 200 nM +
GSK484 20 µM
Supplementary Figure 2. Individual channel images corresponding to the overlays shown in Figure 2. Representative images are presented for each channel: 1) phase-contrast, 2) DNA (green), 3) Annexin V (red), and 4) the composite overlay. Scale bar = 30 µm.

## Slide 3
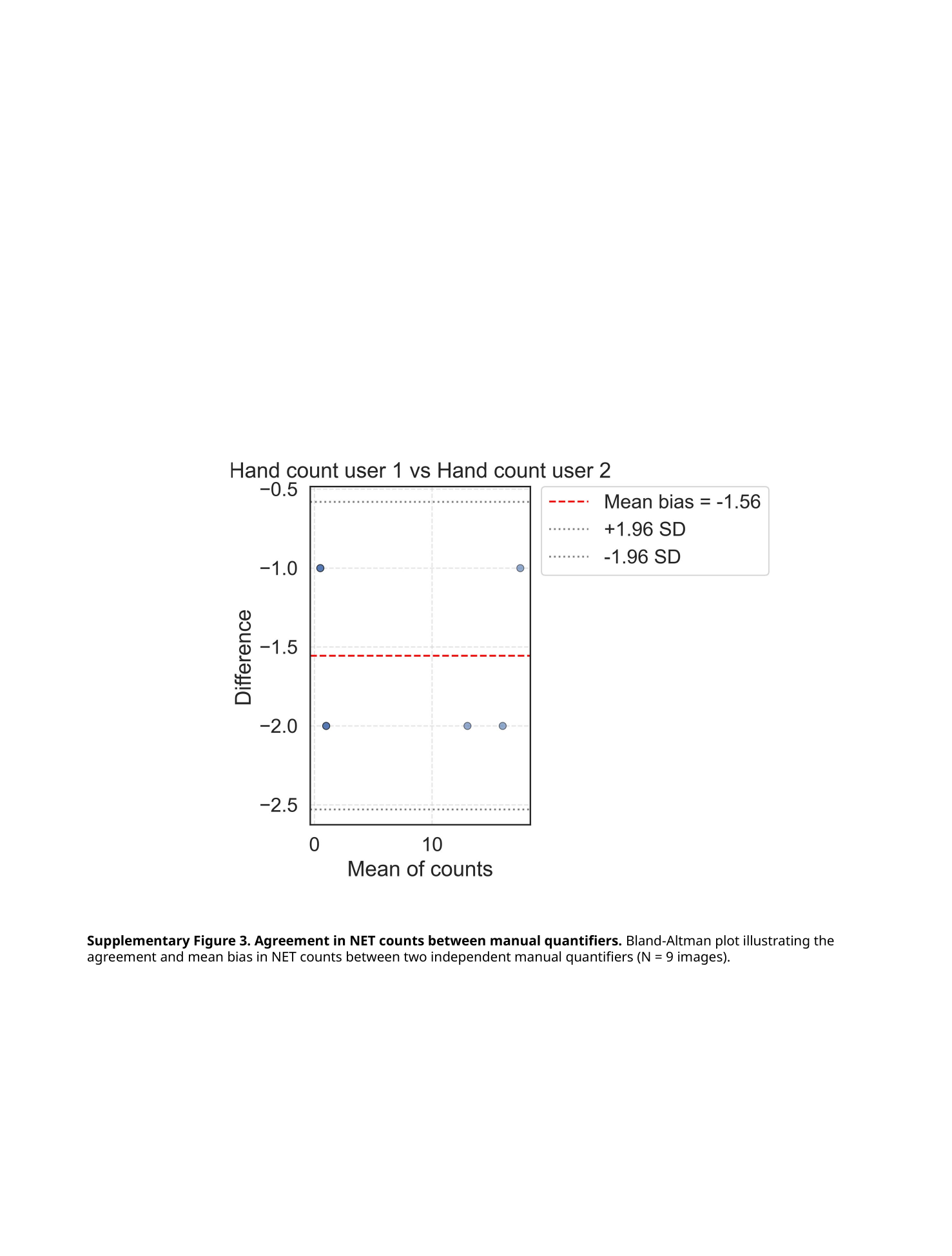

Supplementary Figure 3. Agreement in NET counts between manual quantifiers. Bland-Altman plot illustrating the agreement and mean bias in NET counts between two independent manual quantifiers (N = 9 images).

## Slide 4
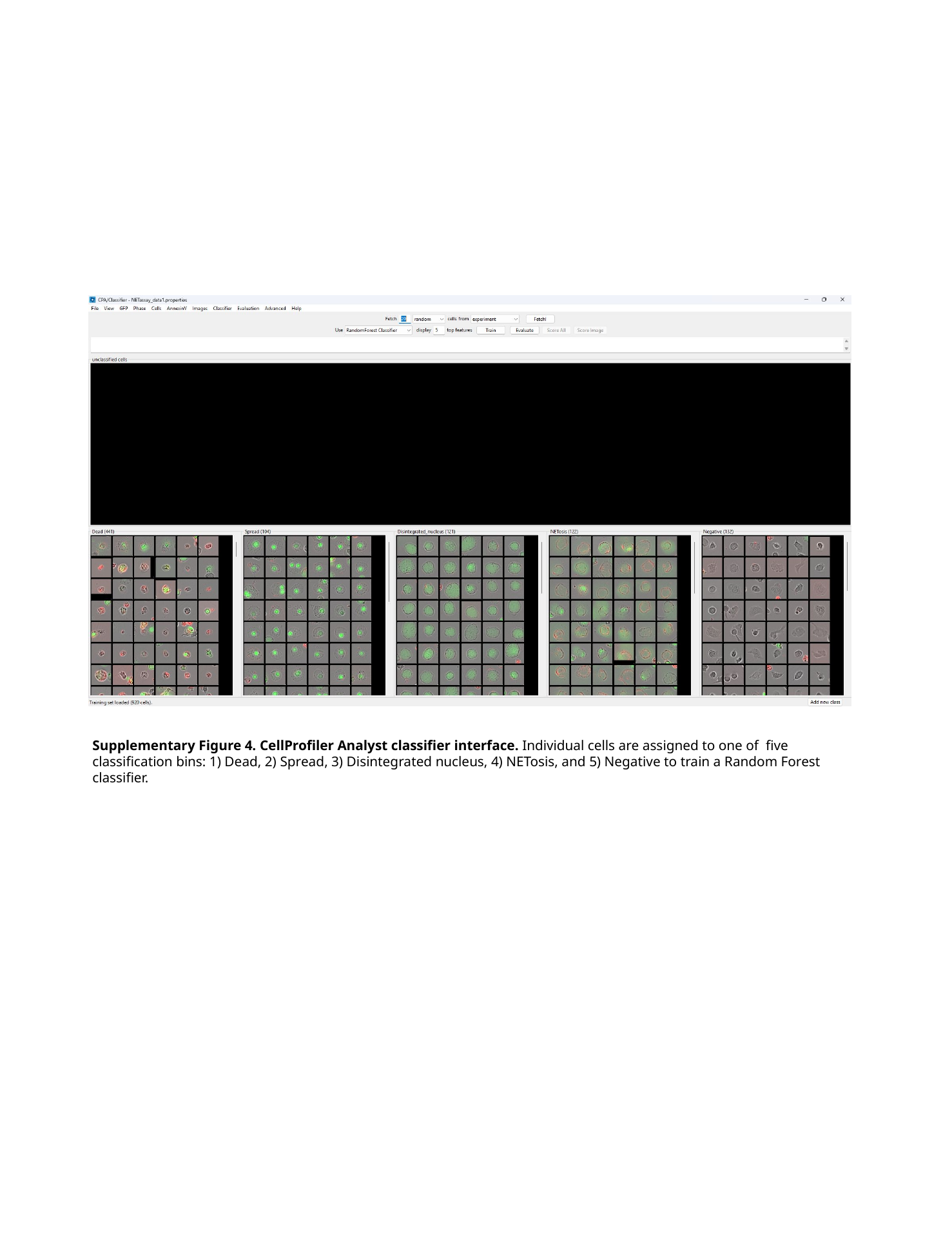

Supplementary Figure 4. CellProfiler Analyst classifier interface. Individual cells are assigned to one of five classification bins: 1) Dead, 2) Spread, 3) Disintegrated nucleus, 4) NETosis, and 5) Negative to train a Random Forest classifier.

## Slide 5
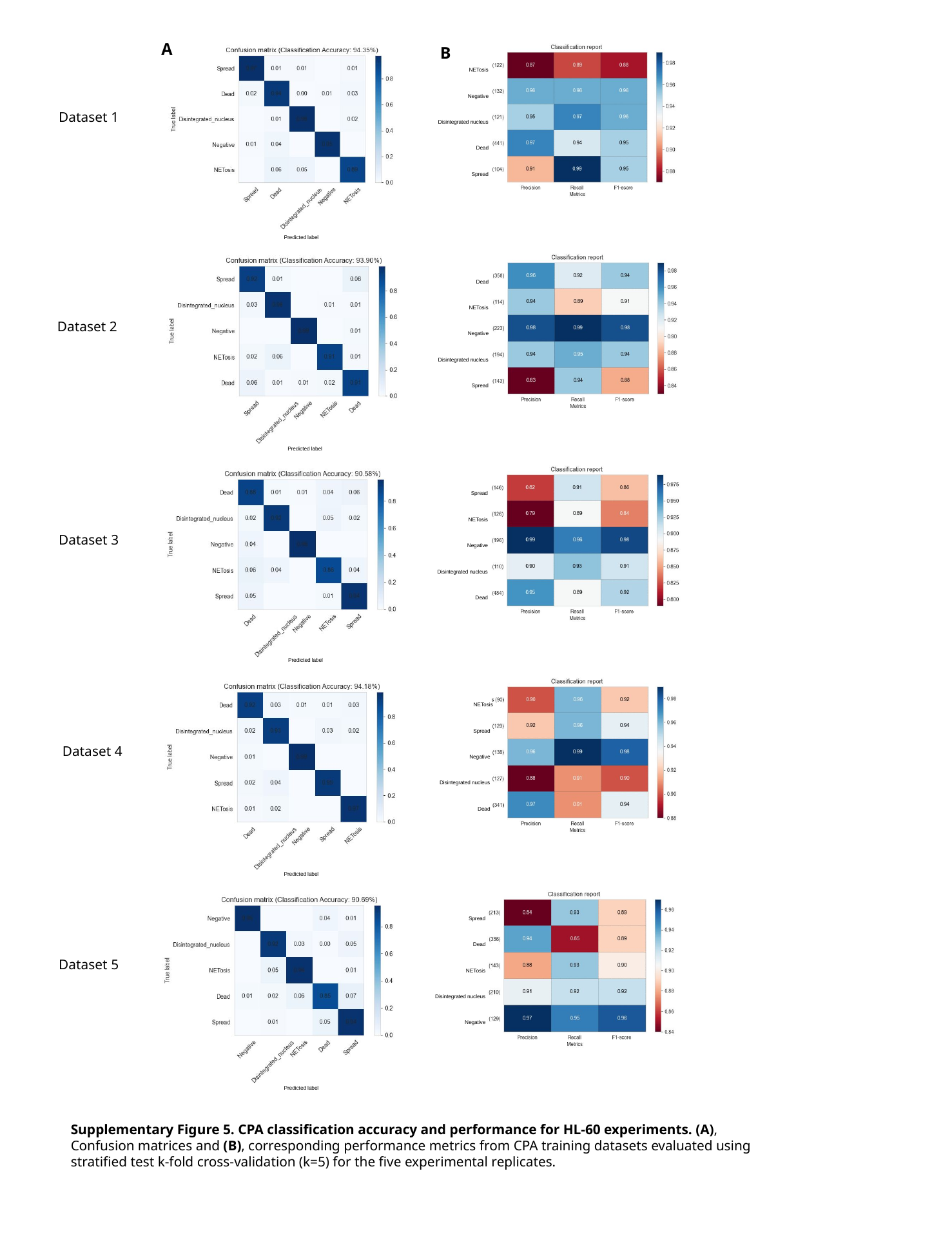

A
B
NETosis
Negative
Disintegrated nucleus
Dead
Spread
Dataset 1
Predicted label
Dead
NETosis
Negative
Disintegrated nucleus
Spread
Dataset 2
Predicted label
Spread
NETosis
Negative
Disintegrated nucleus
Dead
Dataset 3
Predicted label
 NETosis
Spread
Negative
Disintegrated nucleus
Dead
Dataset 4
Predicted label
Spread
Dead
NETosis
Disintegrated nucleus
Negative
Dataset 5
Predicted label
Supplementary Figure 5. CPA classification accuracy and performance for HL-60 experiments. (A), Confusion matrices and (B), corresponding performance metrics from CPA training datasets evaluated using stratified test k-fold cross-validation (k=5) for the five experimental replicates.

## Slide 6
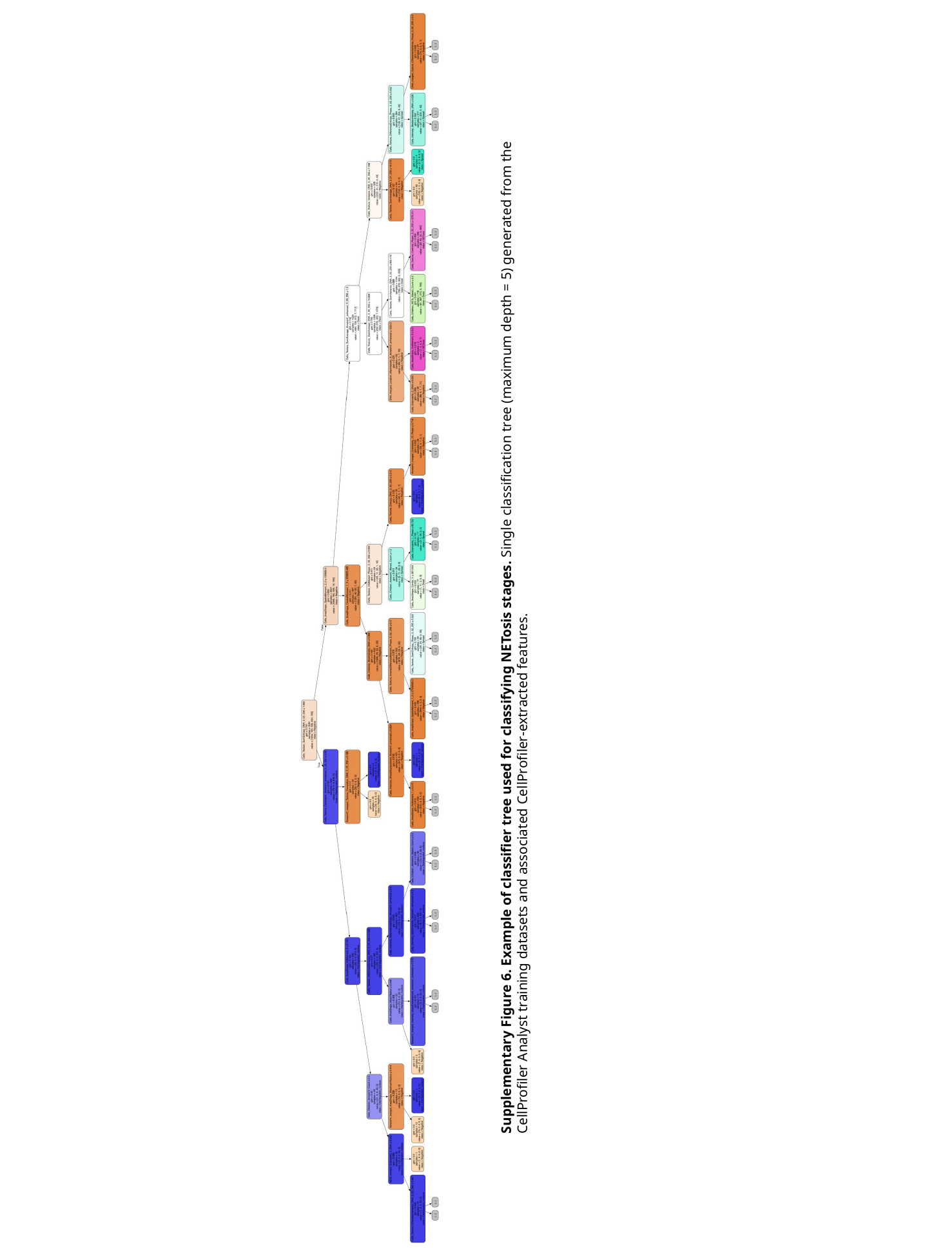

Supplementary Figure 6. Example of classifier tree used for classifying NETosis stages. Single classification tree (maximum depth = 5) generated from the CellProfiler Analyst training datasets and associated CellProfiler-extracted features.

## Slide 7
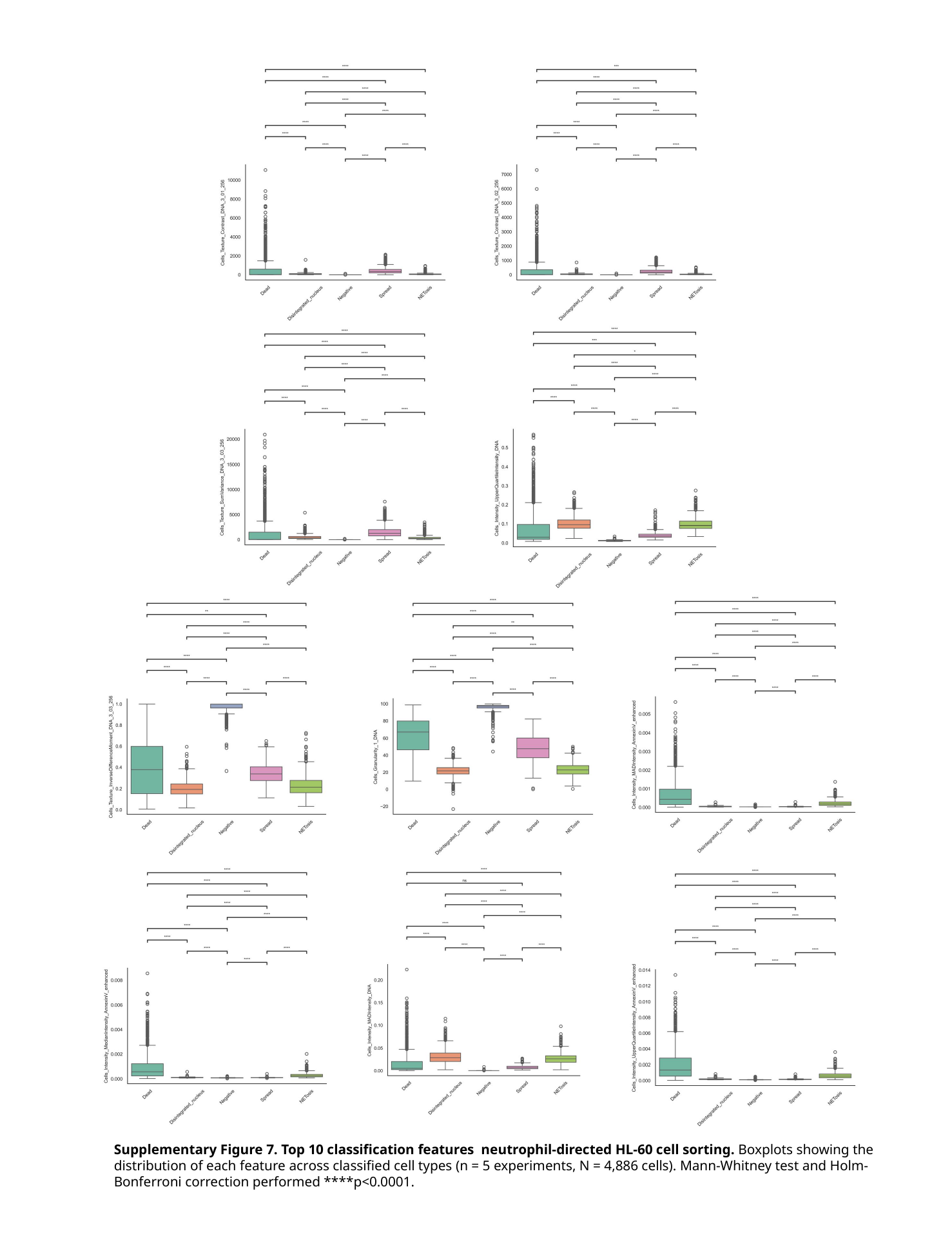

Supplementary Figure 7. Top 10 classification features neutrophil-directed HL-60 cell sorting. Boxplots showing the distribution of each feature across classified cell types (n = 5 experiments, N = 4,886 cells). Mann-Whitney test and Holm-Bonferroni correction performed ****p<0.0001.
