# Supplementary material for: A semi-automated imaging and analysis pipeline for NET quantification and temporal-profiling of NETosis
Source: Front Immunol. 2026 Mar 11;17:1753477. doi: 10.3389/fimmu.2026.1753477 (PMC13012954; doi:10.3389/fimmu.2026.1753477)
Supplement: Supplementary file 3 [file Presentation2.pptx]

## Slide 1
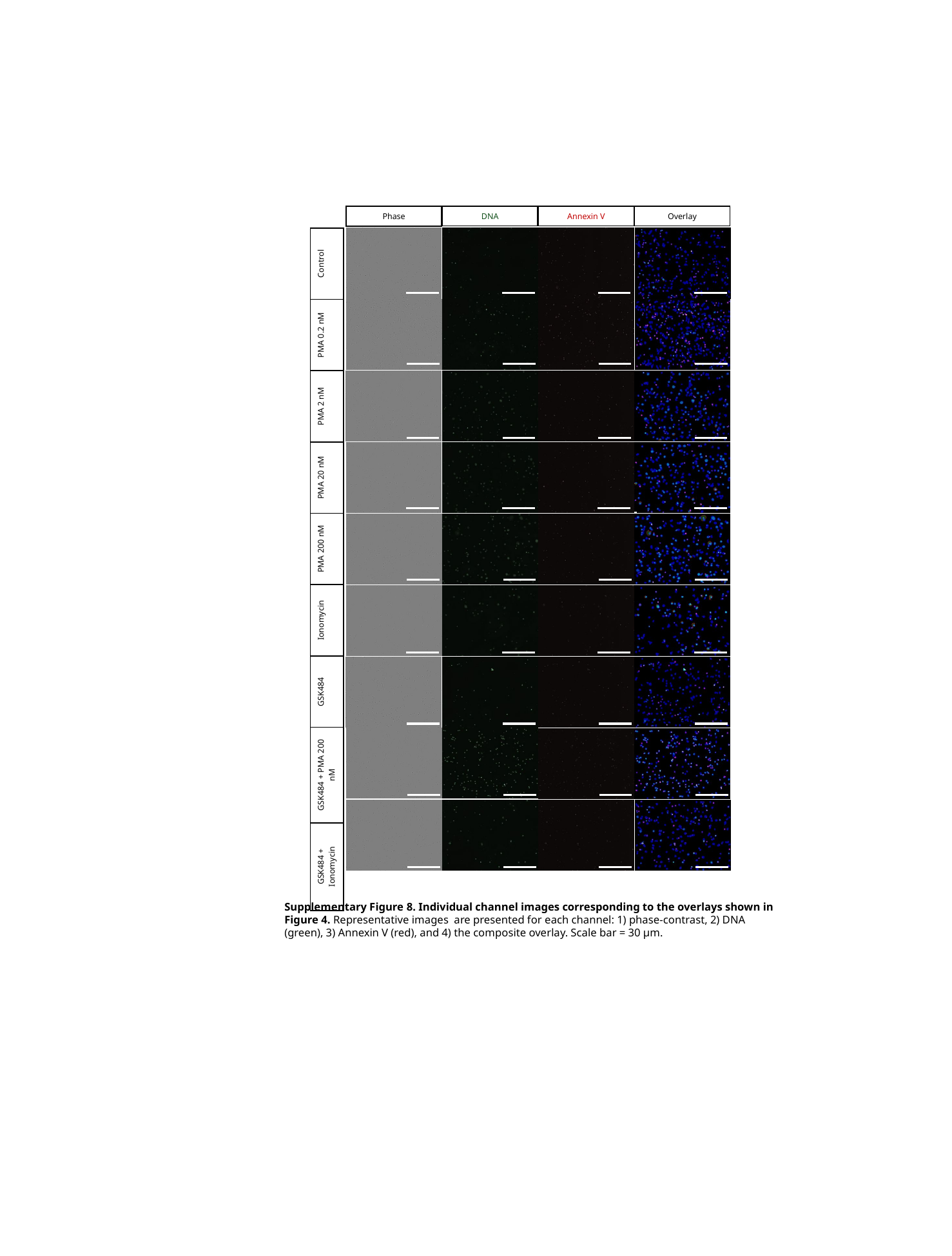

Annexin V
Phase
DNA
Overlay
| Control |
| --- |
| PMA 0.2 nM |
| PMA 2 nM |
| PMA 20 nM |
| PMA 200 nM |
| Ionomycin |
| GSK484 |
| GSK484 + PMA 200 nM |
| GSK484 + Ionomycin |
Supplementary Figure 8. Individual channel images corresponding to the overlays shown in Figure 4. Representative images are presented for each channel: 1) phase-contrast, 2) DNA (green), 3) Annexin V (red), and 4) the composite overlay. Scale bar = 30 µm.

## Slide 2
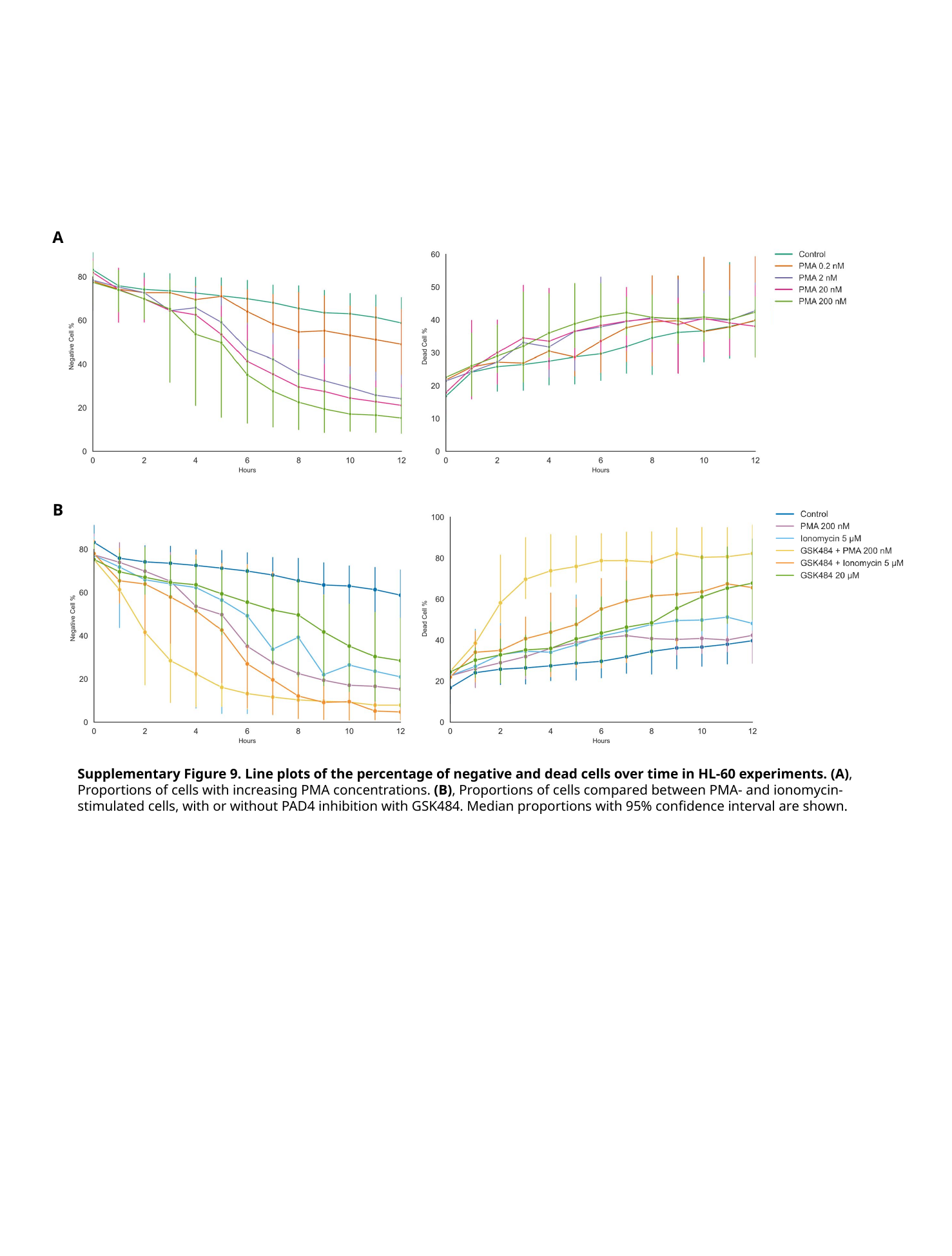

A
B
Supplementary Figure 9. Line plots of the percentage of negative and dead cells over time in HL-60 experiments. (A), Proportions of cells with increasing PMA concentrations. (B), Proportions of cells compared between PMA- and ionomycin-stimulated cells, with or without PAD4 inhibition with GSK484. Median proportions with 95% confidence interval are shown.

## Slide 3
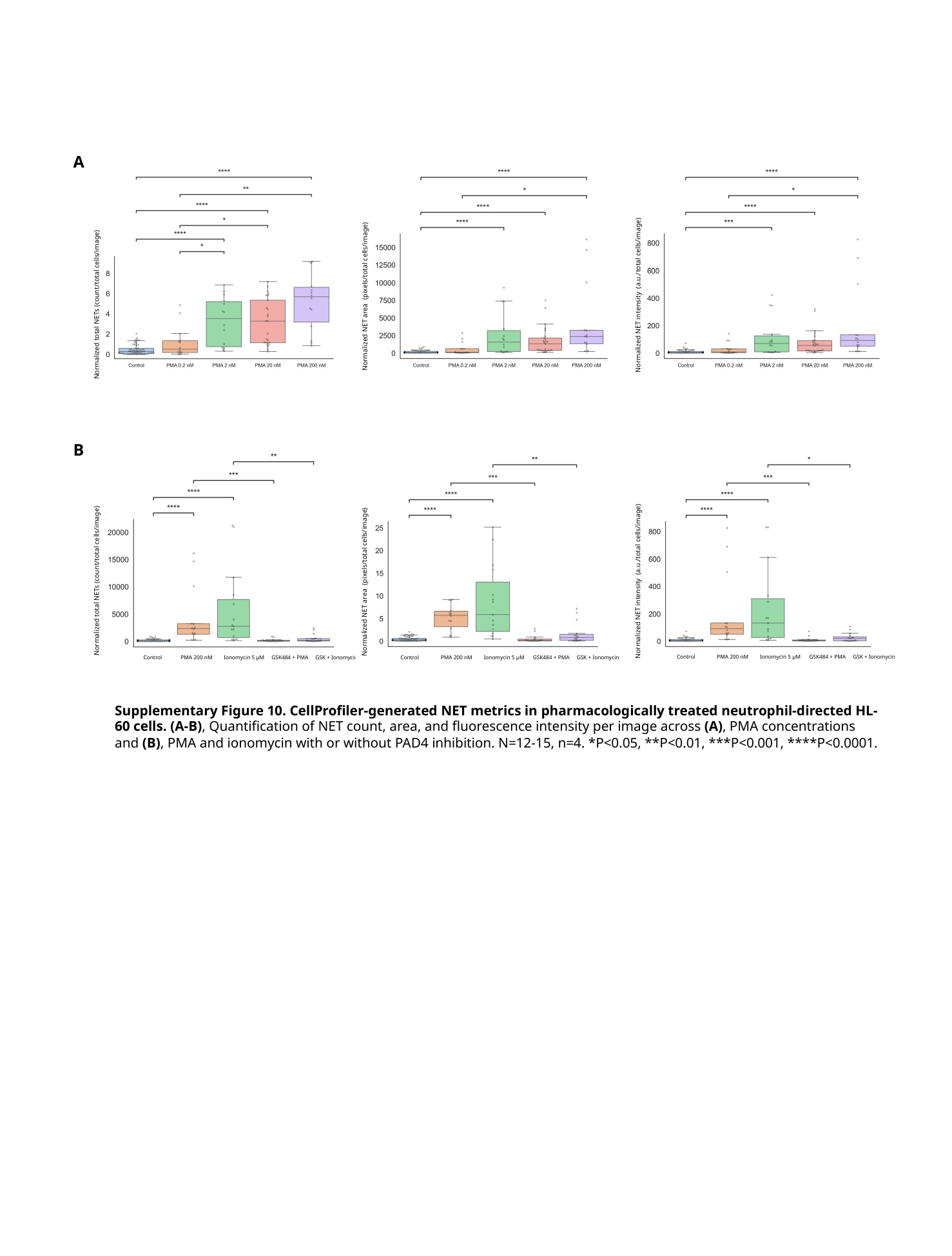

A
Normalized NET intensity (a.u./total cells/image)
Normalized NET area (pixels/total cells/image)
Normalized total NETs (count/total cells/image)
B
Normalized total NETs (count/total cells/image)
Normalized NET intensity (a.u./total cells/image)
Normalized NET area (pixels/total cells/image)
 Control PMA 200 nM Ionomycin 5 µM GSK484 + PMA GSK + Ionomycin
Control PMA 200 nM Ionomycin 5 µM GSK484 + PMA GSK + Ionomycin
Control PMA 200 nM Ionomycin 5 µM GSK484 + PMA GSK + Ionomycin
Supplementary Figure 10. CellProfiler-generated NET metrics in pharmacologically treated neutrophil-directed HL-60 cells. (A-B), Quantification of NET count, area, and fluorescence intensity per image across (A), PMA concentrations and (B), PMA and ionomycin with or without PAD4 inhibition. N=12-15, n=4. *P<0.05, **P<0.01, ***P<0.001, ****P<0.0001.

## Slide 4
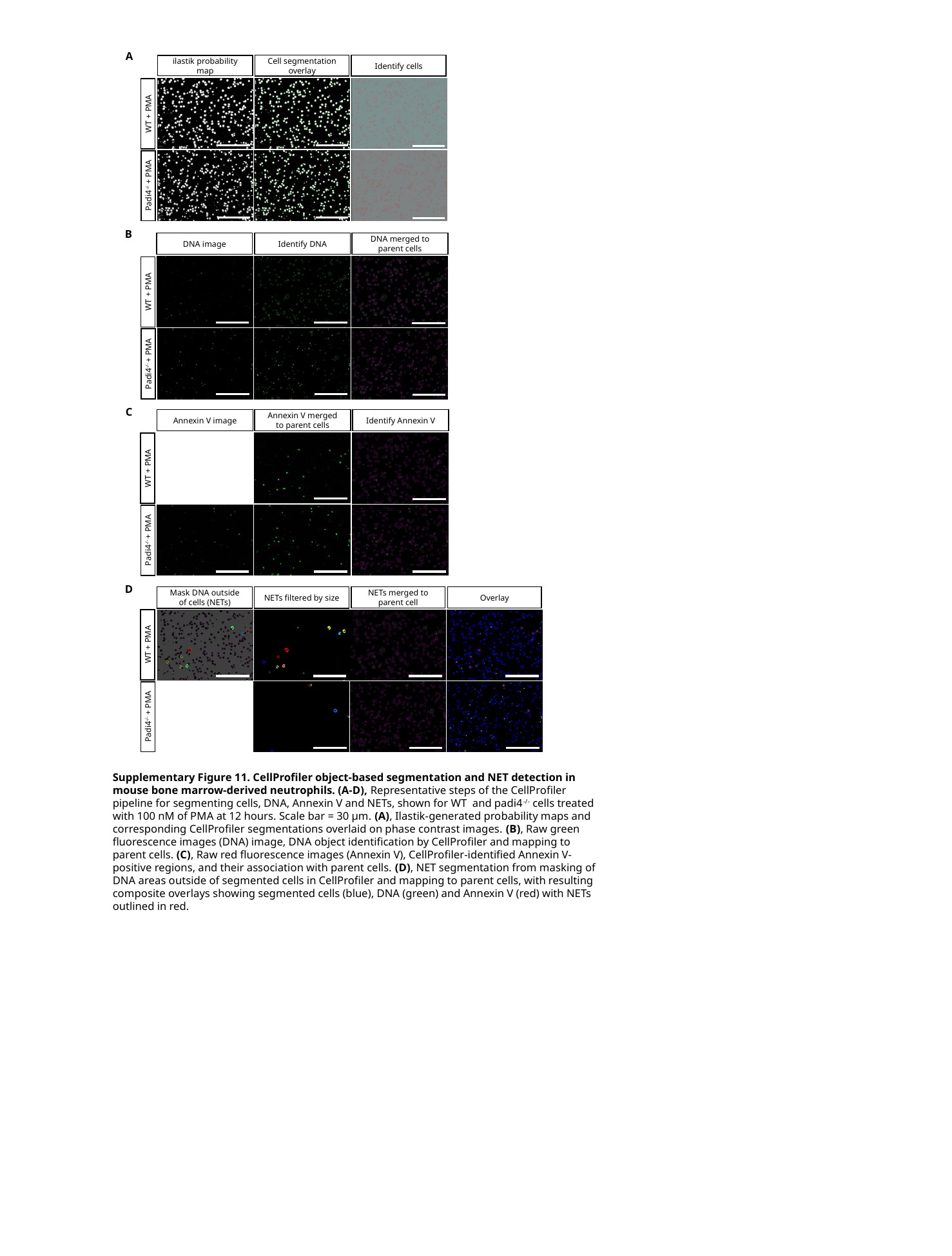

A
Cell segmentation overlay
Identify cells
ilastik probability map
WT + PMA
Padi4-/- + PMA
B
DNA image
Identify DNA
DNA merged to parent cells
WT + PMA
Padi4-/- + PMA
C
Annexin V merged to parent cells
Identify Annexin V
Annexin V image
WT + PMA
Padi4-/- + PMA
D
Mask DNA outside of cells (NETs)
NETs filtered by size
Overlay
NETs merged to parent cell
WT + PMA
Padi4-/- + PMA
Supplementary Figure 11. CellProfiler object-based segmentation and NET detection in mouse bone marrow-derived neutrophils. (A-D), Representative steps of the CellProfiler pipeline for segmenting cells, DNA, Annexin V and NETs, shown for WT and padi4-/- cells treated with 100 nM of PMA at 12 hours. Scale bar = 30 µm. (A), Ilastik-generated probability maps and corresponding CellProfiler segmentations overlaid on phase contrast images. (B), Raw green fluorescence images (DNA) image, DNA object identification by CellProfiler and mapping to parent cells. (C), Raw red fluorescence images (Annexin V), CellProfiler-identified Annexin V-positive regions, and their association with parent cells. (D), NET segmentation from masking of DNA areas outside of segmented cells in CellProfiler and mapping to parent cells, with resulting composite overlays showing segmented cells (blue), DNA (green) and Annexin V (red) with NETs outlined in red.
