# Supplementary material for: A semi-automated imaging and analysis pipeline for NET quantification and temporal-profiling of NETosis
Source: Front Immunol. 2026 Mar 11;17:1753477. doi: 10.3389/fimmu.2026.1753477 (PMC13012954; doi:10.3389/fimmu.2026.1753477)
Supplement: Supplementary file 4 [file Presentation3.pptx]

## Slide 1
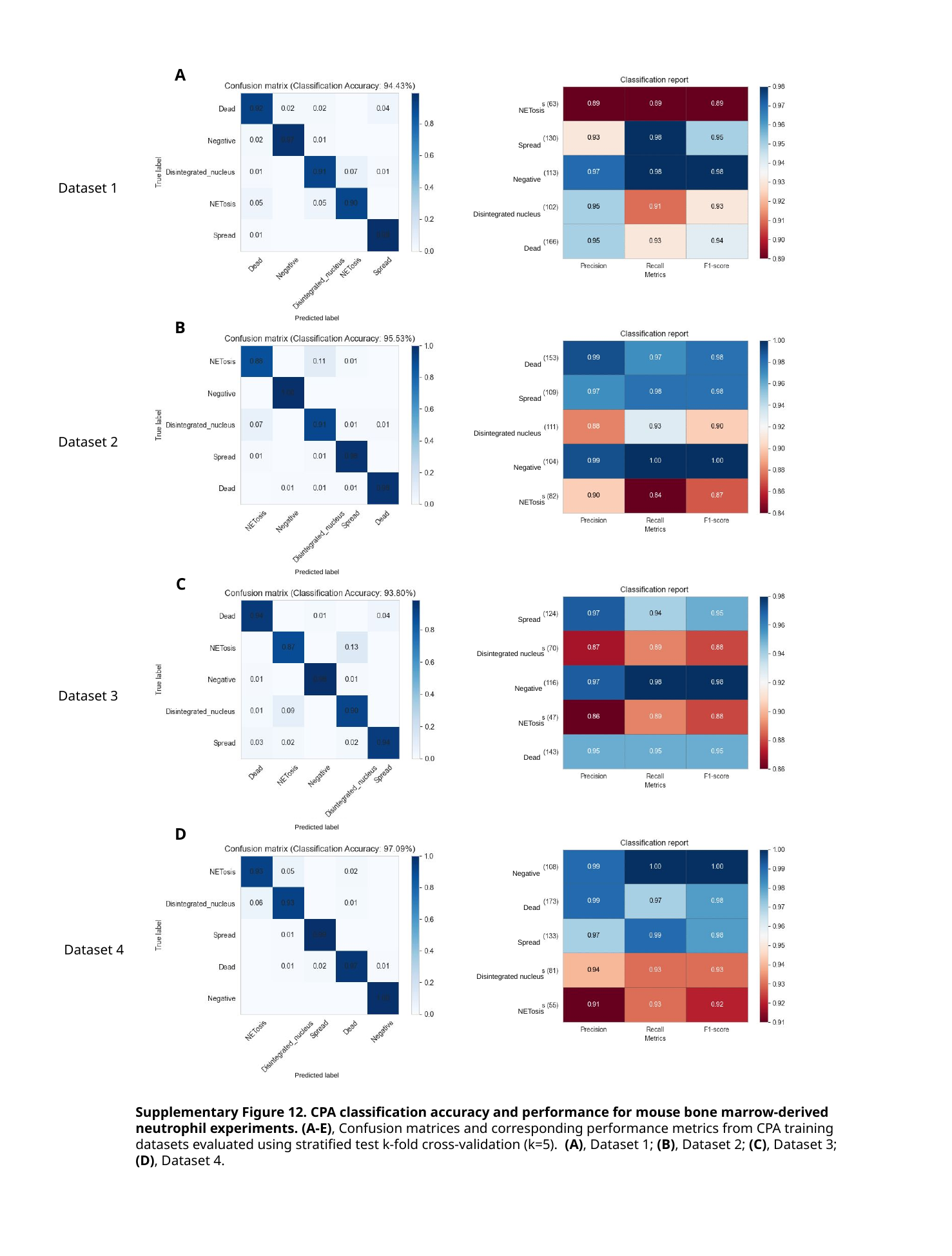

A
NETosis
Spread
Negative
Disintegrated nucleus
Dead
Dataset 1
Predicted label
B
 Dead
 Spread
Disintegrated nucleus
Negative
NETosis
Dataset 2
Predicted label
C
Spread
Disintegrated nucleus
Negative
NETosis
Dead
Dataset 3
Predicted label
D
Negative
Dead
Spread
Disintegrated nucleus
NETosis
Dataset 4
Predicted label
Supplementary Figure 12. CPA classification accuracy and performance for mouse bone marrow-derived neutrophil experiments. (A-E), Confusion matrices and corresponding performance metrics from CPA training datasets evaluated using stratified test k-fold cross-validation (k=5). (A), Dataset 1; (B), Dataset 2; (C), Dataset 3; (D), Dataset 4.

## Slide 2
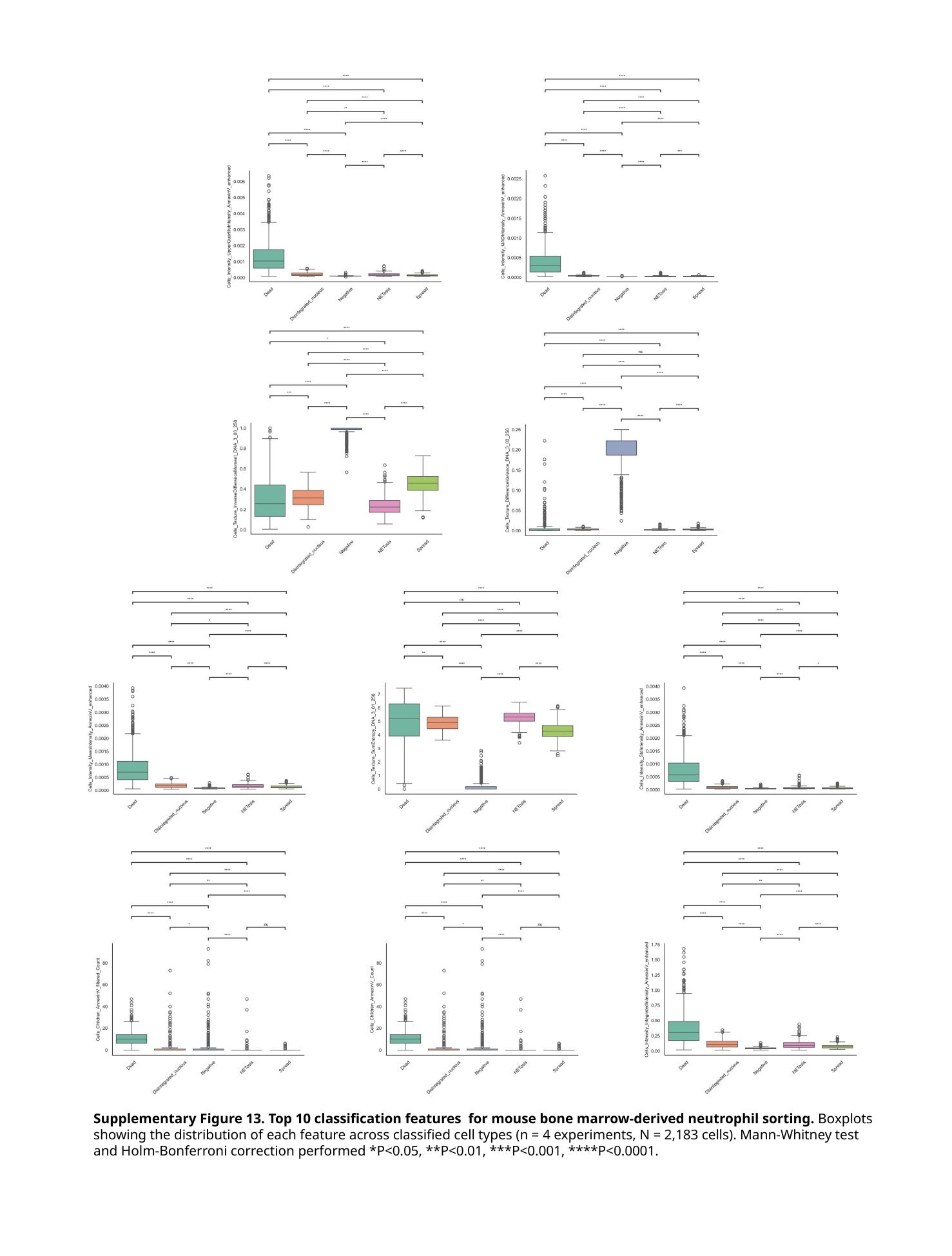

Supplementary Figure 13. Top 10 classification features for mouse bone marrow-derived neutrophil sorting. Boxplots showing the distribution of each feature across classified cell types (n = 4 experiments, N = 2,183 cells). Mann-Whitney test and Holm-Bonferroni correction performed *P<0.05, **P<0.01, ***P<0.001, ****P<0.0001.

## Slide 3
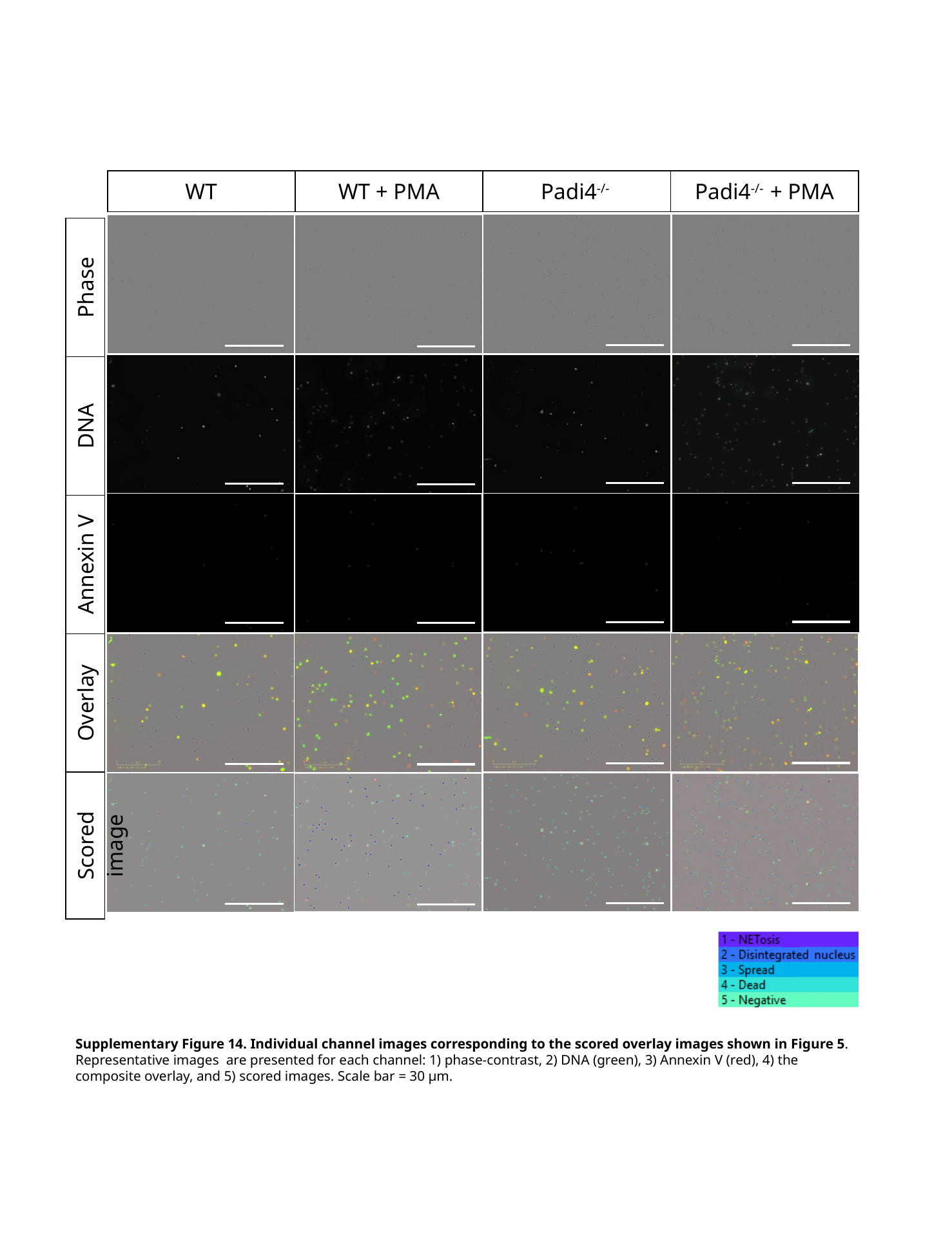

| WT | WT + PMA | Padi4-/- | Padi4-/- + PMA |
| --- | --- | --- | --- |
| Phase |
| --- |
| DNA |
| Annexin V |
| Overlay |
| Scored image |
Supplementary Figure 14. Individual channel images corresponding to the scored overlay images shown in Figure 5. Representative images are presented for each channel: 1) phase-contrast, 2) DNA (green), 3) Annexin V (red), 4) the composite overlay, and 5) scored images. Scale bar = 30 µm.

## Slide 4
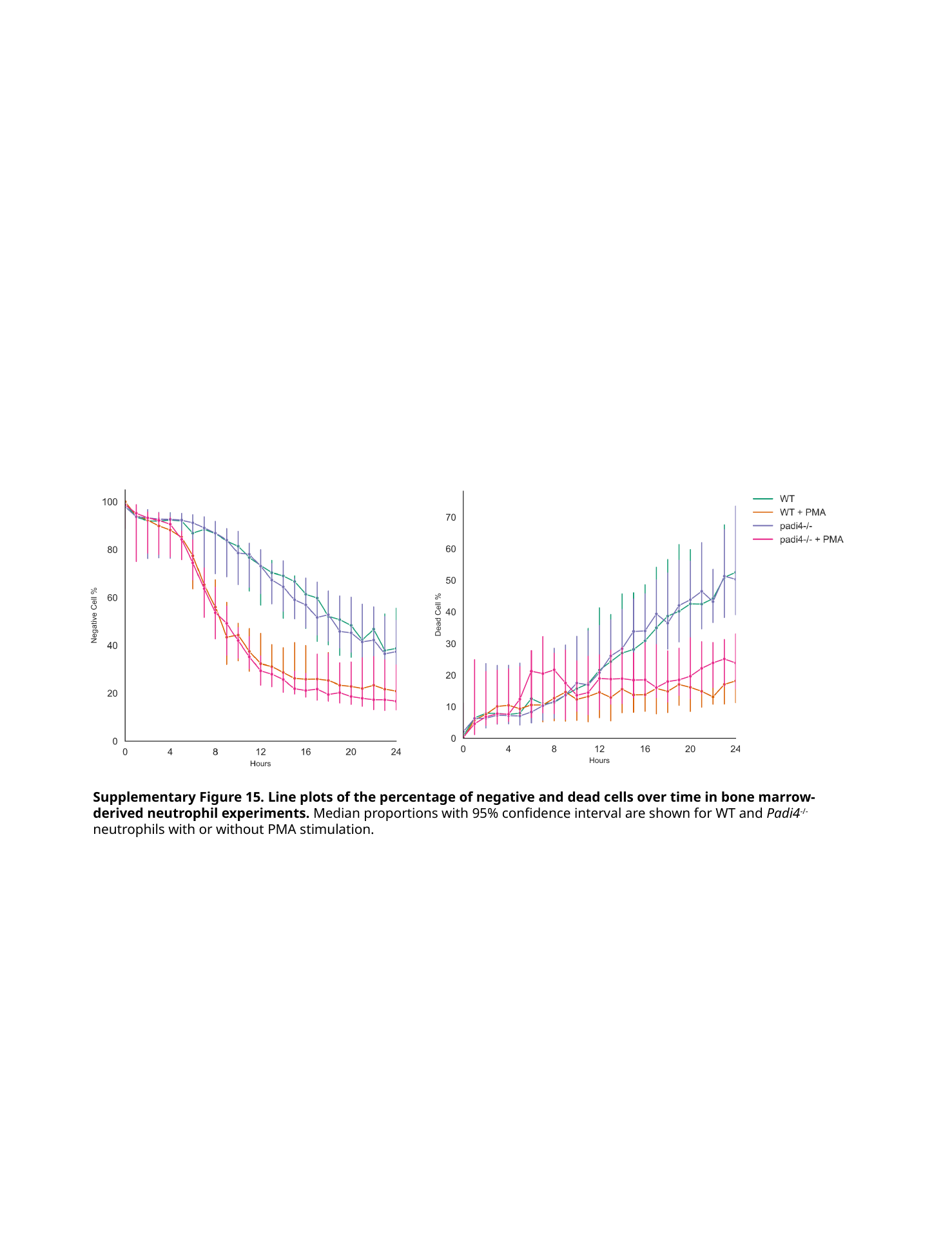

Supplementary Figure 15. Line plots of the percentage of negative and dead cells over time in bone marrow-derived neutrophil experiments. Median proportions with 95% confidence interval are shown for WT and Padi4-/- neutrophils with or without PMA stimulation.

## Slide 5
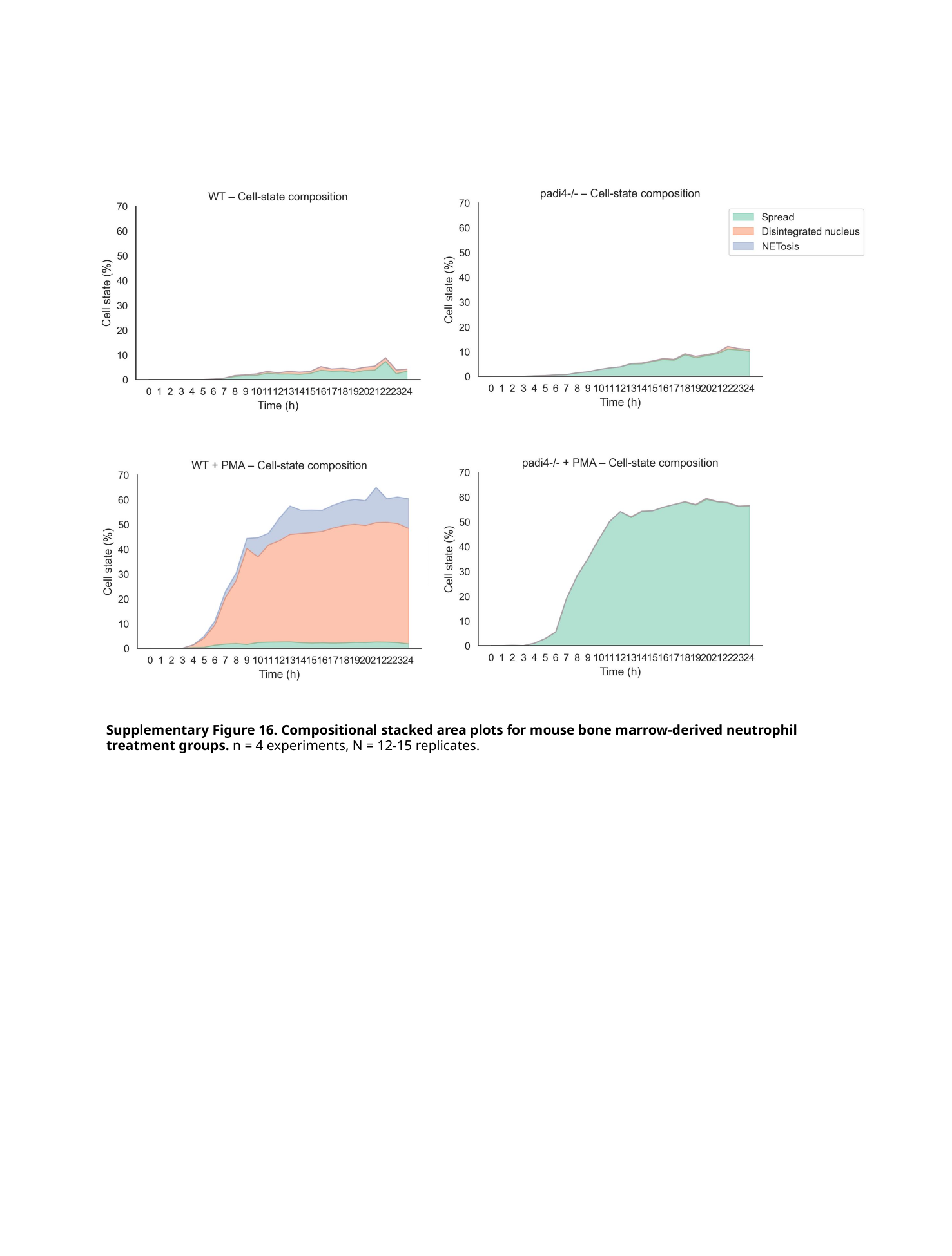

Supplementary Figure 16. Compositional stacked area plots for mouse bone marrow-derived neutrophil treatment groups. n = 4 experiments, N = 12-15 replicates.
